# Evolutionary Conservation and Expression Patterns of Neutral/Alkaline Invertases in *Solanum*

**DOI:** 10.3390/biom9120763

**Published:** 2019-11-21

**Authors:** Luzhao Pan, Qinwei Guo, Songlin Chai, Yuan Cheng, Meiying Ruan, Qingjing Ye, Rongqing Wang, Zhuping Yao, Guozhi Zhou, Zhimiao Li, Minghua Deng, Fengmei Jin, Lecheng Liu, Hongjian Wan

**Affiliations:** 1 College of Horticulture and Gardening, Yangtze University, Jingzhou 434025, China; Lulu_1679@163.com (L.P.); chaisonglina@163.com (S.C.); lchliu18@yangtzeu.edu.cn (L.L.); 2 State Key Laboratory for Managing Biotic and Chemical Threats to the Quality and Safety of Agro-Products, Institute of Vegetables, Zhejiang Academy of Agricultural Sciences, Hangzhou 310021, China; chengyuan1005@126.com (Y.C.); ruanmy@163.com (M.R.); yeqingjing2003@126.com (Q.Y.); rongqingw2012@gmail.com (R.W.); zjyzp@163.com (Z.Y.); Chinazhougz@163.com (G.Z.); zhimiaoli@mail.zaas.ac.cn (Z.L.); 3 Quzhou Academy of Agricultural Sciences, Quzhou 324000, Zhejiang, China; dengmh888@163.com; 4 College of Horticulture and landscape, Yunnan Agricultural University, Kunming 650201, China; kongxonzhu0530@163.com; 5 Tianjin Research Center of Agricultural Biotechnology, Tianjin 300192, China; jinfm414@163.com; 6 China-Australia Research Centre for Crop Improvement, Zhejiang Academy of Agricultural Sciences, Hangzhou 310021, China

**Keywords:** evolution, genome size, sequenced plants, evolutionary time, crystal structures

## Abstract

The invertase gene family in plants is composed of two subfamilies of enzymes, namely, acid- and neutral/alkaline invertases (cytosolic invertase, CIN). Both can irreversibly cleave sucrose into fructose and glucose, which are thought to play key roles in carbon metabolism and plant growth. CINs are widely found in plants, but little is reported about this family. In this paper, a comparative genomic approach was used to analyze the CIN gene family in *Solanum*, including *Solanum tuberosum*, *Solanum lycopersicum*, *Solanum pennellii*, *Solanum pimpinellifolium,* and *Solanum melongena*. A total of 40 CINs were identified in five *Solanum* plants, and sequence features, phylogenetic relationships, motif compositions, gene structure, collinear relationship, and expression profile were further analyzed. Sequence analysis revealed a remarkable conservation of CINs in sequence length, gene number, and molecular weight. The previously verified four amino acid residues (D188, E414, Arg430, and Ser547) were also observed in 39 out of 40 CINs in our study, showing to be deeply conserved. The CIN gene family could be distinguished into groups α and β, and α is further subdivided into subgroups α1 and α2 in our phylogenetic tree. More remarkably, each species has an average of four CINs in the α and β groups. Marked interspecies conservation and collinearity of CINs were also further revealed by chromosome mapping. Exon–intron configuration and conserved motifs were consistent in each of these α and β groups on the basis of *in silico* analysis. Expression analysis indicated that CINs were constitutively expressed and share similar expression profiles in all tested samples from *S. tuberosum* and *S. lycopersicum*. In addition, in CIN genes of the tomato and potato in response to abiotic and biotic stresses, phytohormones also performed. Overall, CINs in *Solanum* were encoded by a small and highly conserved gene family, possibly reflecting structural and functional conservation in *Solanum*. These results lay the foundation for further expounding the functional characterization of CIN genes and are also significant for understanding the evolutionary profiling of the CIN gene family in *Solanum*.

## 1. Introduction

Sucrose (D-glucopyranosyl-(1–2)-D-fructofuranose), as the principal end product of photosynthesis in higher plants and photosynthetic bacteria, is translocated through the phloem from the source tissue into sink organs as a transport molecule [1,2,3]. Sucrose is either separated into simple hexoses (glucose, galactose, and mannose), or degraded into derivatives, such as deoxy sugar, to supply carbon and energy or to act as signaling molecules for plant growth, development, and stress tolerance [4,5,6,7]. A growing body of evidence indicated that sucrose and its hydrolysis products play an essential role in signal transduction, osmotic adjustment, and gene expression for plant growth and development [5,6,8,9]. 

Sucrose is cleaved to hexose by two prime enzymes: sucrose synthase (SuS; EC 2.4.1.13) and invertase (INV; EC 3.2.1.26). The sucrose molecule is cleaved by SuS into uridine diphosphate-glucose and fructose in a reversible manner, whereas it is irreversibly hydrolyzed by INV into fructose and glucose [4,5]. Researchers suggested that SuS is mainly involved in the biosynthesis of sugar polymers, such as starch, cellulose, and energy (ATP) generation [10]. Similarly, INV also widely participates in the regulation of plant growth and development, including controlling reproductive development and responses to stress tolerance [4,7,11,12]. Particularly, it was found that the activity of cytosolic invertase was indispensable to the growth and reproduction of *Arabidopsis thaliana* [13]. 

Invertases are composed of two gene families on the basis of their optimal pH: alkaline/neutral invertases with pH optima between 6.5 and 8.0, and acid invertases with pH optima between 4.5 and 5.0 [14]. Usually, alkaline/neutral invertases are targeted to the cytoplasm and other organelles including mitochondria and plastids (cytosolic invertase, CIN), belonging to glycoside hydrolase family 100 (GH100), whereas acid invertases pertain to the GH32, located in either the cell-wall matrix or vacuoles (CWIN and VIN, respectively) [4,14]. Both CWINs and VINs share high sequence similarity, and are closely related to each other in the phylogenetic tree. Moreover, acid invertases can hydrolyze sucrose and other β-fructose-containing oligosaccharides, such as raffinose, stachyose, and 1-kestose; hence, they are also named β-fructofuranosidases [14]. On the contrary, CINs specifically catalyze sucrose, and are different from acid invertases on many levels. In addition, it was generally believed that acid invertases originated from respiratory eukaryotes and aerobic bacteria, while CINs were speculated to stem from cyanobacteria [15].

Functional characterization or regulatory mechanisms for acid invertases have been widely reported in the last few decades. For example, VINs not only regulate floral organ differentiation and the formation of seeds in cotton, but play leading roles in sugar accumulation and cell expansion [11,16]. In addition, much evidence indicated that CWINs and VINs are conducive to defense responses to abiotic and biotic stresses [3,10,17,18]. In contrast to CWINs and VINs, less is known about CINs’ physiological function or regulatory mechanism due to their low and labile activity [4]. Moreover, CINs were once thought to be ‘cinderella’ enzymes for a long time, i.e., not involved in sucrose metabolism and plant development [1,10].

However, exciting studies are constantly emerging that offer new insights into the elusive enzyme. Up to now, CINs have been studied in a limited number of regulation functions, such as gene expression, root growth, and response to diverse environmental stresses [12,13,19,20]. Recently, the regulation of CIN activity (AtCINV1) was thought to be mediated by protein phosphorylation in the post-translational level in *Arabidopsis* [21]. They identified a residue, Ser547 of *AtCINV*1, at the extreme C-terminus, which is a substrate of calcium-dependent kinases 3 and 21, and that the phosphorylation of Ser547 allowed a high-affinity binding site with 14-3-3 proteins. Interestingly, the family of 14-3-3 proteins, as a conserved regulatory molecule, was found in all eukaryotic cells, and it could be activated by light, leading to enhanced CIN activity in the roots [21]. 

In addition, analysis of the crystal structures of CINs (InvA and InvB) in a cyanobacterium revealed overall structural similarity of InvA and InvB invertases [22,23]. They share an (α/α)6-barrel core structure composed of 12 α-helices and an insertion of three helices. Two putative catalytic residues, Asp188 and Glu414 in InvA, and InvA’ stringent substrate specificity towards the α1, 2-glycosidic bond of sucrose were further proposed [22]. In InvB, the Arg430 residue possesses different conformation, likely facilitating the deprotonation of catalytic residue Glu415 [23]. CINs are evolutionarily and functionally more stable compared with CWINs and VINs according to the phylogenetic relationship and functional genomic analysis, which may reflect their roles in maintaining cytosolic sugar homeostasis for cellular function [24]. 

To date, CIN gene families have been investigated in several plant species, such as *Oryza sativa*, *Arabidopsis*, *Capsicum annuum*, *Populus trichocarpa*, *Manihot esculenta*, *Vitis vinifera*, and *Lotus japonicus*, as well as *Hevea brasiliensis* [20,25,26,27,28]. These previous studies have expanded our knowledge on the CIN gene family and presented several common characteristics. For example, the CIN gene family can be distinguished into two distinct groups, namely, the α and β subgroups; CIN genes from the α group differ in gene structure from those of the β group; CIN genes of the β group are localized in the cytosol, and α CINs are localized in plastids or mitochondria. 

Here, to investigate the functional and structural characterizations of CINs, systematically comparative genome analysis was performed on the CIN gene family in five *Solanum* plant species, namely, *Solanum tuberosum*, *Solanum lycopersicum*, *Solanum pennellii*, *Solanum pimpinellifolium,* and *Solanum melongena*. On the basis of the bioinformatics method, we characterized sequence features, phylogenetic relationship, chromosomal localization, exon–intron structures, and expression profiles. These analyses provide valuable information for elucidating the evolutionary relationship and function of the CIN gene family in the near future.

## 2. Materials and Methods

### 2.1. Identification of CIN Genes in Solanum

All protein sequences from five *solanum* species were downloaded from three public databases: *S. tuberosum* from the Potato Genome Sequencing Consortium (PGSC, http://solanaceae.plantbiology.msu.edu/pgsc_download.shtml); *S. pennellii*, *S. lycopersicum,* and *S. pimpinellifolium* from the Sol Genomics Network (SGN, Release 2.5, https://solgenomics.net/); and *S. melongena* from the Eggplant Genome DataBase (ftp://ftp.kazusa.or.jp/pub/eggplant/). Subsequently, a local database was constructed by employing Bioedit 7.0 software [29]. The Hidden Markov Model (HMM) profile of the Glyco_hydro_100 (GH100) conserved domain (PF12899) was downloaded from the Pfam protein family database (http://pfam.sanger.ac.uk/). The AT1G35580 gene in *Arabidopsis* was considered as a neutral/alkaline invertase [21]. We searched the amino acid sequence of the AT1G35580 gene using the NCBI website (https://www.ncbi.nlm.nih.gov/). A BlastP search was carried out to identify CIN genes across five plant species using AT1G35580 as a query, and the E-value was set at e^−5^. All predicted CIN genes were initially identified, and these candidates were then sent to the Pfam database to examine the existence of the GH100 domain. The protein sequences with conserved domain were retained, and sequences with a partial domain were manually removed. 

The protein length and molecular weight of the CIN gene family were calculated by using the ProtParam server (http://web.expasy.org/protparam/). Chromosome location, genomic sequences, coding sequences (CDS), and intron numbers were retrieved from the SGN online database (http://solgenomics.net/search/locus). The divergence dates of *Solanum* plants were also calculated using a website (http://www.timetree.org/).

### 2.2. Multiple Sequence Alignment and Phylogenetic Analysis

Multiple sequence alignment of each amino acid sequence from five *Solanum* species was conducted using the ClustalX1.83 software with default settings [30]. Then, similar protein sequences were highlighted with different colors using the Bioedit 7.0 software. On the basis of the sequence-alignment results, a maximum-likelihood phylogenetic tree was constructed by using MEGA7.0 tool [31]. The identity level of the CIN gene family was calculated by using the DNAMAN software (Lynnon Biosoft, San Ramon, CA, USA).

### 2.3. Conserved Motifs and Gene Structure of CIN Genes

The MEME server (http://meme-suite.org/tools/meme) and Tbtools were employed to analyze the motif composition of CINs [32]. A total of 20 different motifs, namely, motifs 1 to 20, were acquired. Intron–exon distribution and intron-phase patterns were illustrated by aligning the cDNA sequences and genomic sequences of CIN genes by using the online Gene Structure Display Server 2.0 (http://gsds.cbi.pku.edu.cn/).

### 2.4. Mapping CIN Members on Chromosomes

CIN genes mapped on the chromosomes of three species (*S. lycopersicum*, *S. pennellii*, and *S. tuberosum*) were visualized in the MapDraw 2.1 software [33]. The tandem duplicated gene pairs were determined on the basis of previously reported methods [34,35], and confirmed with the following criteria: (1) an array of 2 or more CIN candidates within a range of 100 kb distance; (2) alignment had a coverage rate of more than 70% of that of the longer gene; and (3) the identity of the aligned region was no less than 70%. Segmental duplication was investigated on the online Plant Genome Duplication Database (PGDD, http://chibba.agtec.uga.edu/duplication/).

### 2.5. Tissue-Specific Expression of CINs in Tomato and Potato

Data concerning CIN gene transcription were extracted from the tomato functional genomics database (TFGD, http://ted.bti.cornell.edu/) and the PGSC (http://solanaceae.plantbiology.msu.edu/), respectively. The Fragments Per Kilobase Million mapped reads (FPKM) values of CIN genes were submitted to the MultiExperiment Viewer 4.9 (MeV4.9) software and then log_2_-transformed [36]. Two heat maps of the CIN gene family in different tissue types in the tomato and potato were obtained. 

### 2.6. Expression Profiling of CINs in Response to Different Stresses

In order to investigate the expression profiles of the CIN gene family in the potato plant, all available data of the regulating factor were downloaded from the PGSC (http://solanaceae.plantbiology.msu.edu/). The expression values of CIN genes were processed as log_2_ transformation fold change in control vs. treatments. Heat maps were created by using MeV4.9 [34]. The expression value of each CIN gene that showed more than twofold up- or downregulation (*p* < 0.05) was regarded as differently expressed.

### 2.7. Plant Materials and Stress Treatment 

Plants of the tomato cultivar (zhefen702), produced by the Zhejiang Academy of Agricultural Sciences, were used in our experiment. Seeds were placed on water-saturated filter paper to be germinated. These seedlings, with a condition of 25/18 °C 12/12 h day/night cycle, were planted in a growth chamber. At the age of 4–6 of fully expanded young tomato leaves, the tomato plants were treated under chemical stresses with gibberellins (GA_3_; 100 μM), 6-benzylaminopurine (6-BA) (100 μM), salicylsalicylic acid (SA) (100 μM), abscisic acid (ABA) (100 μM), naphthylacetic acid (NAA; 100 μM), and jasmonic acid (JA; 100 μM). Leaf samples were harvested at 0, 1.5, 3, 6, 12, and 24 h after treatments, respectively. These collected samples were immediately frozen with liquid nitrogen for total RNA extraction. We performed three independently biological replications on each sample type.

### 2.8. Total RNA Extraction and Quantitative Real-Time PCR (qRT-PCR)

Total RNA was isolated using a Total RNA kit (Tiangen Biotech, Beijing, China), and the quality and concentration of RNA were evaluated by ultra-micro-nucleic acid protein tester (NanoDrop One). Reverse transcription was further conducted using a FastQuant RT Kit (Tiangen Biotech, Beijing, China) on the basis of the manufacturer’s procedure. A total of 20 μL volume, containing 6.8 μL of distilled water, 10 μL of 2× SYBR Green PCR MasterMix, 1.2 μL of each primer, and 2 μL of template (10× diluted cDNA from samples), was included for qRT-PCR analysis. QRT-PCR reactions were carried out using an ABI StepOne real-time fluorescence quantitative PCR instrument (Applied Biosystems, Foster City, CA, USA). Melt curves were used to evaluate the quality and specificity of primers. Three technical replicates were conducted for each of the CIN genes. The expression values of CIN genes were calculated by the 2^−ΔΔct^ method [37].

## 3. Results and Discussion

### 3.1. Conservation in CIN Gene Family: Gene Number, Sequence Length, and Molecular Weight 

Neutral/alkaline invertases were identified in *Solanum*, including *S. tuberosum*, *S. lycopersicum*, *S. pennellii*, *S. pimpinellifolium*, and *S. melongena*, which made it possible to perform comparative genomic analysis for the CIN gene family. A total of 58 putative CINs were preliminarily predicted in these five fully sequenced plant genomes (Table 1). Among the 58 sequences, however, each of the six CIN genes from the three species, namely, *S. lycopersicum*, *S. pimpinellifolium*, and *S. melongena*, was manually excluded because of the absence of a significant portion of their coding regions, which probably encode truncated proteins, or could be nonfunctional or pseudogenes. Twelve genes existing as alternative splicing in the potato genome were also eliminated. Thus, 18 redundant CIN members from four different species were filtered out. In the end, a total of 40 CIN sequences from *Solanum* were selected for further analysis. Interestingly, each of the five *Solanum* plants possesses eight CIN genes. Previous studies have suggested that seven to eleven CINs were identified through a systematic survey in several plant genomes (such as maize, *Arabidopsis*, rice, pepper, and cassava) [26,27], whereas these were eight in all the surveyed *Solanum* species, indicating stability in the number of this small gene family among various plant species. 

**Figure 1:**
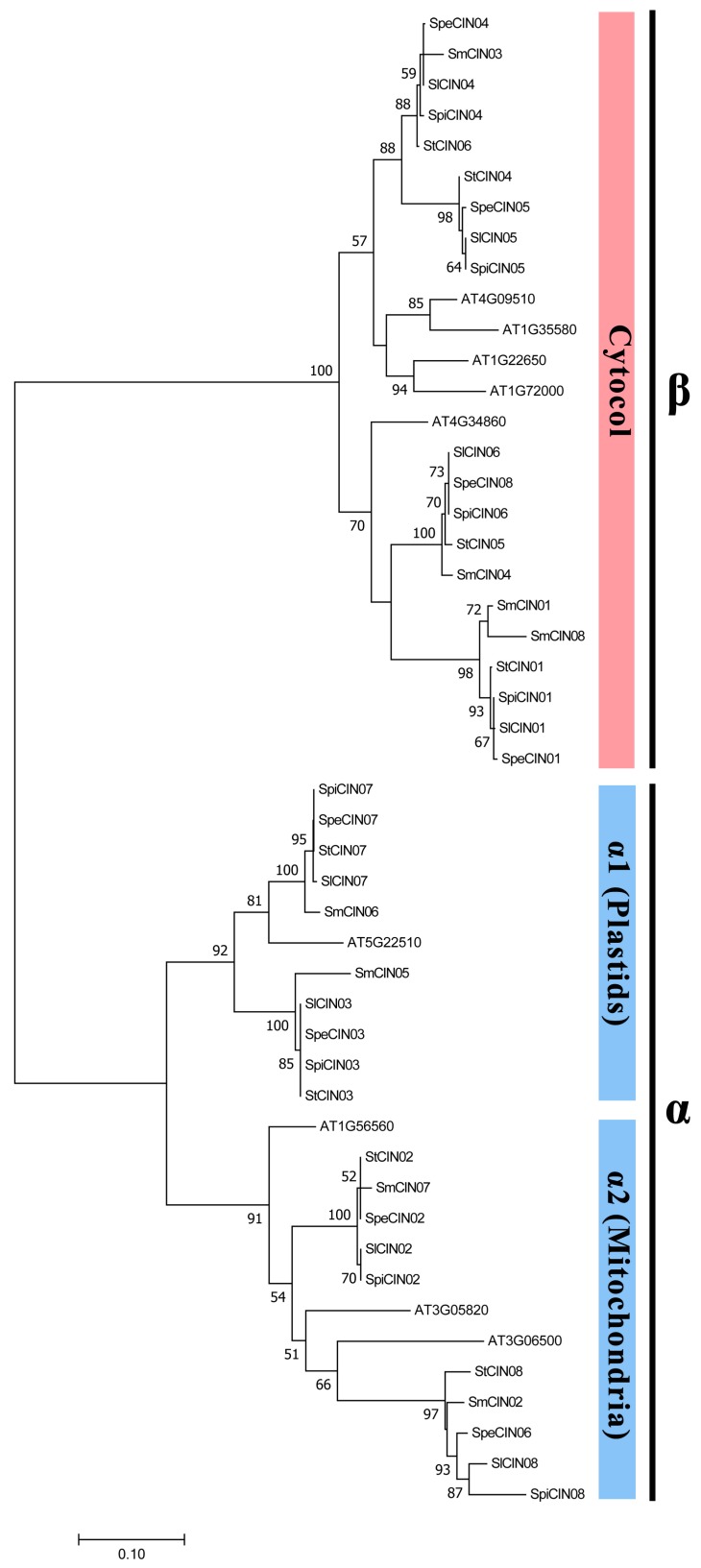
Phylogenetic relationship of *Solanum* CINs with *Arabidopsis*. Phylogenetic tree of CIN proteins was constructed with the maximum-likelihood method using MEGA 7.0 software. Support values shown in each line in the phylogenetic tree. Putative *CIN* genes divided into two subfamilies (α and β) on the basis of their *in silico* prediction of subcellular localization; the α subfamily was further divided into α1 and α2.

The CIN gene family in plants can be classified into two major classes, the α and β subfamilies, on the basis of their sequence homology [20,25,26,27,28]. Combined with currently phylogenetic analysis, we found that the protein length and molecular weight of CIN genes dramatically varied between the α and β groups, but they were relatively stable in the corresponding group (Figure 1 and Table S1). In the α group, protein length ranged from 547 (*SmCIN*07) to 678 (*StCIN*08) amino acids (AAs), and molecular weight was between 69.1 (*SmCIN*07) and 76.6 (*StCIN*08) kD, with a mean of 643 AAs and 73kD (Table S1). In the β group, variations of protein length were from 412 (SmCIN04 and SpeCIN08) to 605 (*SpiCIN*06) AA, and molecular weight from 47.3 to 69.1 kD, with a mean of 543 protein sequences and 62 kD (Table S1). Of these finally selected 40 CIN genes, *SmCIN*08 belonging to the β group only encoded 249 amino acid residues, which is possibly due to a genomic sequencing error or annotation error (Table S1). Therefore, shorter sequences from eggplant genome were manually eliminated from this statistical analysis. On the basis of length and molecular weight, the CIN gene family was well-conserved in the corresponding group.

### 3.2. Potential Functional Conservation: Four Key Amino Acid Residues 

**Figure 2:**
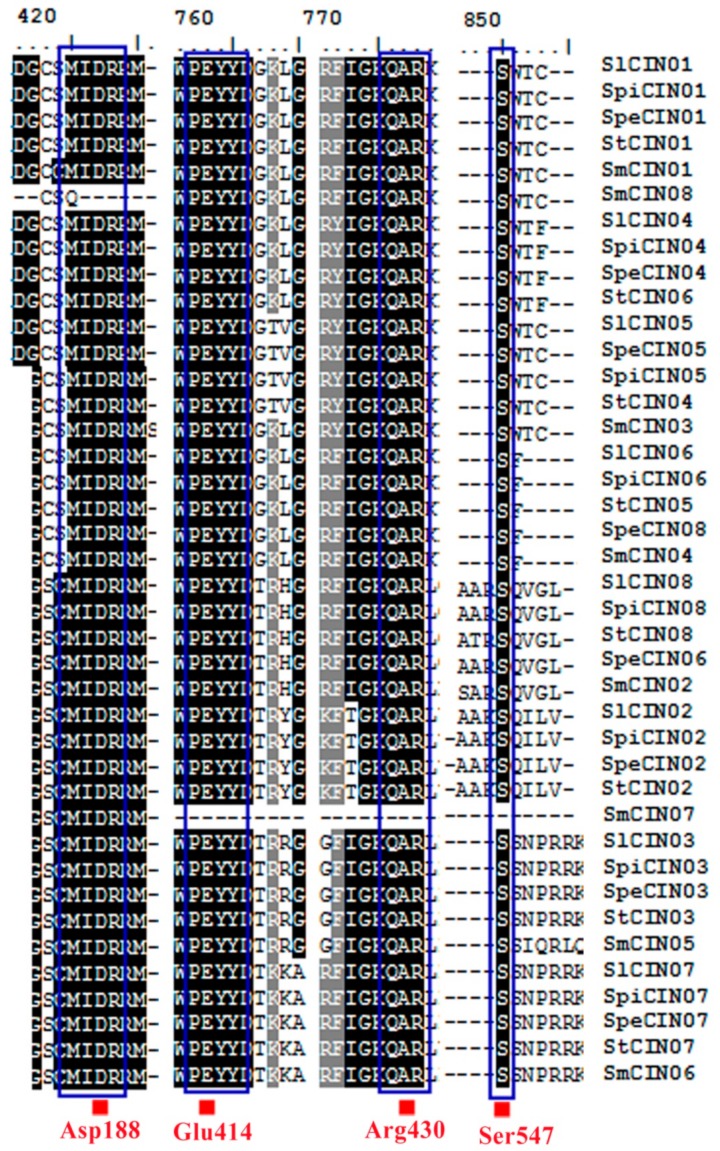
Conserved amino acid residues of CIN genes in *Solanum*. Species abbreviations: Sl: *Solanum lycopersicum*, Spi: *Solanum pimpinellifolium*, Spe: *Solanum pennellii*, St: *Solanum tuberosum*, Sm: *Solanum melongena*. Names of all CIN members listed on figure’s right side. Dark shading reflects 100% amino acid residue conservation. Four conserved catalytic residues (Asp188, Glu414, Arg430, Ser547) depicted by blue squares.

It was recently reported that three amino acid residues (D188, E414, and Arg430) were found to be associated with catalytic activity, established in crystal-structure comparison and enzymatic assays of CINs (InvA and InvB), which is isolated from the cyanobacterium *Anabaena* [22,23]. In the current paper, these three conserved residues were also observed in all CIN genes within five *Solanum* plants on the basis of sequence alignment, except that SmCIN08 was missing D188, and SmCIN07 lacked the E414 and Arg430 residues (Figure 2 and Table S1). Phosphorylation of an amino acid residue (Ser547) in the CIN gene family by calcium-dependent kinases enables binding to 14-3-3 proteins to raise CIN activity [21]. In our study, residue Ser547 was extensively detected at the extreme C-terminus of CIN protein sequence in all of the analyzed CIN genes except for SmCIN07. More interestingly, between these four completely conserved amino acid residues, three of them are localized in the C-terminal portion of CIN genes (Table S1). Thus, we speculated that the C-terminus of the CIN gene family tends to have higher sequence similarity than the N-terminal region, and C-terminal sequences might be involved in the catalytic activity or substrate binding of CINs.

It was believed that shared sequence fragments harbor conserved functions and structural attributes that could also reveal previously undiscovered protein families, as well as new members to existing ones [38]. In this study, we further analyzed the sequence traits of CIN genes by multiple sequence alignment. These CIN genes shared a large number of the same fragments or a high degree of sequence homology, especially in the C-terminal region (Table S2). These results revealed the conservation of the structure and function of the CIN gene family. In addition, the similarity level of the *CIN* genes showed that the lowest and highest identity were 43.1% and 100%, respectively, and average pairwise identity was 70.86% (Table S3). We also found a higher average pairwise identity for CIN members in the β group than that of those in the α group. The former was 90.55%, ranging from 80.5% to 100%, whereas the latter was 72.89%, ranging from 42.6% to 100%. This result indicated that these *CIN*s in the β group possessed higher sequence identity compared with the α group members. In other words, the β group is ascribed to have higher conservation, and it is possible to reflect functional conservation for β *CIN*s in a common ancestor.

### 3.3. Evolutionary Conservation of CIN Genes Based on Phylogenetic Relationship

**Figure 3:**
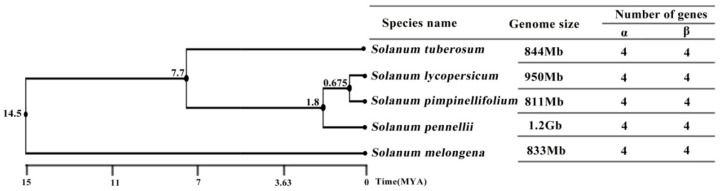
Evolutionary time, genome size, and numbers of CIN genes within each plant species. Evolutionary time in million years ago (MYA) shown in each line, and scale shown at the bottom of the species tree.

To clarify the evolutionary relationship of the CIN gene family, *Solanum* CINs were aligned with members of *Arabidopsis* CINs, and then maximum-likelihood (ML) and maximum-parsimony (MP) phylogenetic trees were constructed using MEGA7.0 (Figure 1). These two methods essentially gave the same trees except for some minor details (Figure S1). According to the dendrogram, these genes could be separated into two primary groups, corresponding to the α and β groups, and the α group was further divided into the α1 and α2 subgroups. Interestingly, between the α and β group, each conformably included four CIN members that derived from each out of five species (Figure 1 and Figure 3). These results revealed that the CIN gene family was also extremely conserved in terms of the number of intragroup genes. The phylogenetic separation of thee α and β groups is statistically supported by bootstrap analysis and an exon–intron structure, and classification is in exact accordance with earlier reports on this topic [20,25,26,27,28]. Hence, our phylogenetic analysis for the CIN gene family has high confidence. Analyses also showed that the CIN gene family from the β group had higher sequence similarity compared with the α group within *Solanum* from the point of view of the bar scale in the phylogenetic tree (Figure 2). 

The β group *CIN*s were identified to be cytosol-targeted, while the latter were inferred to be organelle-targeted (such as plastids or mitochondria) [39]. Our results and previous reports further hint that the *CIN* genes of cytosol might be more stable than *CIN* genes in organelles in the evolution of the *Solanum* plants, possibly suggesting that cytosolic CINs are more conserved than organelle-localized CINs in the α group.

We further studied the evolutionary time between five wholly sequenced plants, and searched the genome size of each species on the basis of a previous report [40]. A species tree and the number of genes were determined between the α and β groups in each plant (Figure 3). The eggplant, with approximately 1.3 Gb genome size, appeared earliest between five species, and divergence date was about 14.5 million years ago (MYA). The potato and three tomato species were separated by 7.7 MYA, and the potato genome was estimated to be around 0.84 Gb. The three tomato species are *S. pennellii*, which emerged approximately 1.8 MYA with a 1.2 Gb genome; and *S. lycopersicum* and *S. pimpinellifolium*, which diverged 0.7 MYA, and have 0.95 and 0.8 Gb genomes, respectively. These results showed a large span of species-differentiation time in these five Solanum genomes, but their genomic size was relatively stable. More interestingly, the same number of CINs was found in each *Solanum* species, and an equal number of CIN genes were present in each species of the α and β group (Figure 1 and Figure 3), suggesting that the CIN gene family was stable and did not expand during the evolution of *Solanum* plants. It was also shown that gene number is not directly related to the genome size of the CIN gene family that was conserved throughout *Solanum* evolution, despite a previous author making the claim that gene number and genome size are actually correlated [41].

### 3.4. Conservation in CIN Motif Compositions and Gene Structures 

**Figure 4:**
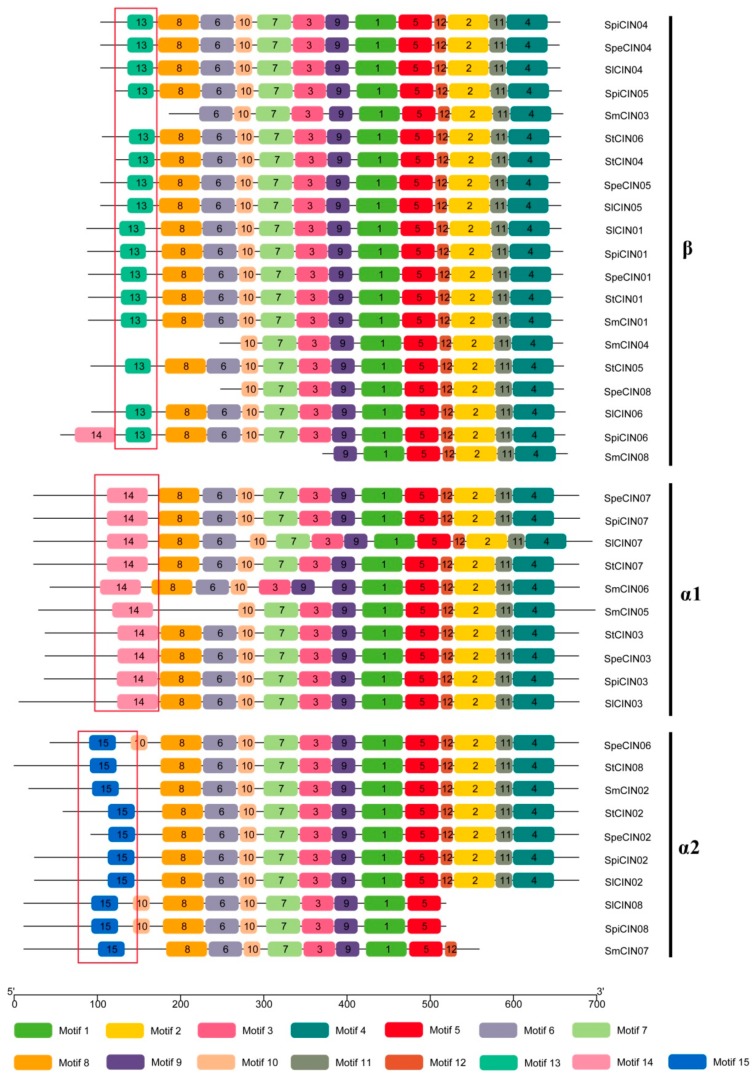
Conserved motifs of 40 CIN proteins. Seven CIN genes (*SmCIN*03/04/07/08, *SpeCIN*08 *SlCIN*08, *SpiCIN*08) are missing some conserved motifs in the N- or C- terminus due to containing shorter amino acids. Differently colored boxes represent different types of motifs. Specific motifs in β, α1, and α2 subgroups are labeled with red boxes.

With the aim of determining motif types and locations, we carried out an investigation of conserved motifs in each of the CIN proteins out using MEME and TBtool search software. A total of 15 distinct motifs were identified in the CIN gene family in *Solanum*, and the presence of the motifs in representative CINs from different subgroups is provided in Figure 4. Most CIN genes comprise a mass of completely conserved motifs. For example, 32 out of 40 CIN genes (80%) share 12 identical motifs, namely, motifs 8, 6, 10, 7, 3, 9, 1, 5, 12, 2, 11, and 4. That is to say, CIN genes within the α and β groups appeared highly conserved on the whole motif level in *Solanum*. 

Relatively poor conservation was also observed in two subgroups (α1 and α2) among the CIN genes, showing subgroup-specific intergroup features. Three diverse motifs (motifs 13–15) were unique to each subgroup (Figure 4). Motif 13 is unique to β CIN members in the phylogenetic tree, while motifs 14 and 15 are specific to α1 and α2 CINs, respectively. These motifs were used to distinguish between the β, α1, and α2 subgroups. Overall, our analysis suggested the CIN gene family was highly conserved, and intergroup specificity was also observed during the evolution process of the *Solanum* species. These CIN proteins contain completely conserved motifs within each of these two groups, which may potentially share similar functional characteristics [42]. On the contrary, functional differentiation also occurred between the α and β groups of the CIN gene family [39]. However, the function of these highly conserved or intergroup-specific motifs still needs further studies in the near future.

We also found that α CINs have longer protein sequences than those of β CINs in the N-terminal region (Figure 4), whereas N-terminal sequences experienced larger sequence divergence with a longer C-terminal than that of the β group (Table S1). This led us to speculate that CINs of the α group may retain more signal peptide sequences in the process of plant evolution, which is probably the reason for the multilocalization of α CINs.

**Figure 5:**
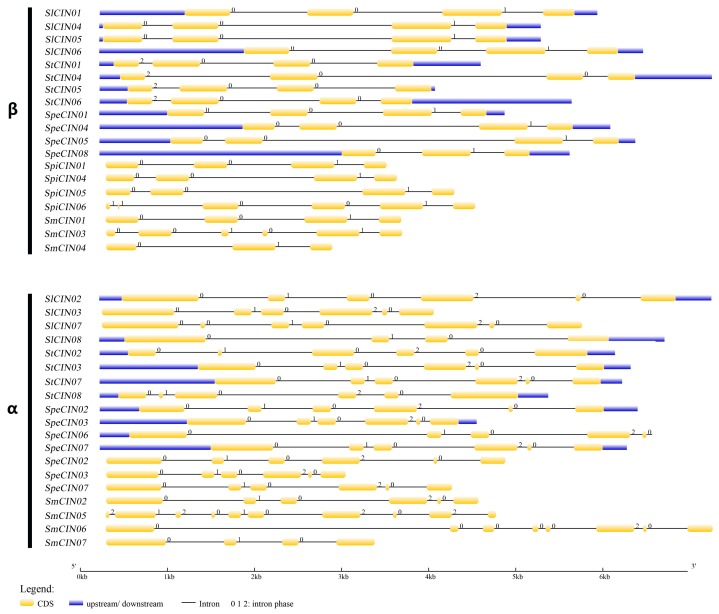
Exon–intron structure of 38 CINs in *Solanum*. Introns shown as black lines, and exons shown as yellow boxes. Upstream or downstream untranslated sequences represented by blue boxes. Phase 0 represents intron located between two codons; phase 1 represents intron inserted into the first base of a codon; phase 2 represents intron inserted/interrupt into the second base of a codon. Scale is shown at the bottom of the diagrams.

Many genes in the CIN gene family are typically encoded by four or six exons in different classes [25,26,27]. In our paper, two groups of CIN genes in five different plants also differed in exon features (Figure 5). In line with previous reports, CINs from β group usually possess four exons, and α CIN members are encoded by six exons. Within the β subfamily, 15 CIN genes consist of four exons, while the four remaining members (SpeCIN08, SpiCIN06, SmCIN03, and SmCIN04) encoded irregular exons (Figure 5). The intron phases of these β CIN members showed a 0-0-1 phase pattern, but four CINs from the potato genome presented a 2-0-0 pattern. Within the α branch, a total of 15 CIN members were predicted to be encoded by six exons (excluding SlCIN07/08 and SmCIN05/06/07), and the intron phase showed 0-1-0-2-0. Interestingly, each of the 14 CIN genes contained one shorter exon, as if it was erroneously predicted by an intron or had originated within other exons. The integrated gene-structure models of *CIN*s in *Solanum* were very similar to those of CIN members from pepper, grapevine, cassava, and rice, as reported previously [25,26,27,43]. On the basis of the exon–intron structure and intron-phase pattern, the CIN gene family in *Solanum* was highly conserved in each corresponding group. That led us to suspect that the CIN gene family from the α and β groups had functional differentiation occurring during evolution. 

Taken together, analysis of the conserved motifs and gene structures again shed light on high conservation among *CIN* genes in *Solanum*, which could be used to verify functional conservation. Highly conserved motifs and group-specific gene structures were also in accordance with the results of phylogenetic analysis and classification mentioned above.

### 3.5. Strong Observed Collinearity in CIN Gene Family

**Figure 6:**
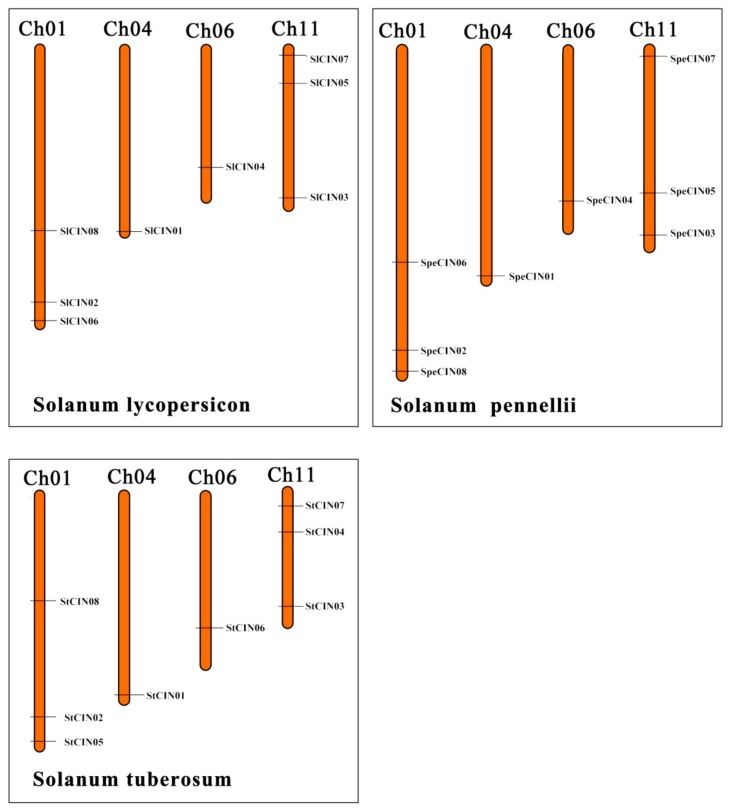
Chromosome localization of CIN genes from *S. lycopersicum*, *S. pennellii,* and *S. tuberosum*. CIN gene positions shown as black lines. Chromosome numbers shown at the top of each bar.

After the evaluation of the motifs and gene structure, we continued our analysis with a focus on the chromosomal distribution of CINs in three species, *S. lycopersicum*, *S. pennellii*, and *S. tuberosum*. As shown in Figure 6, a total of 24 CIN members (each species contained eight genes) were randomly distributed on their chromosomes. Chromosomes 01 and 11 from each of three species harbored three CIN genes, while chromosomes 04 and 06 of each species had only one orphan CIN. We also found that most CIN genes are placed on the distal ends of chromosomes.

**Figure 7:**
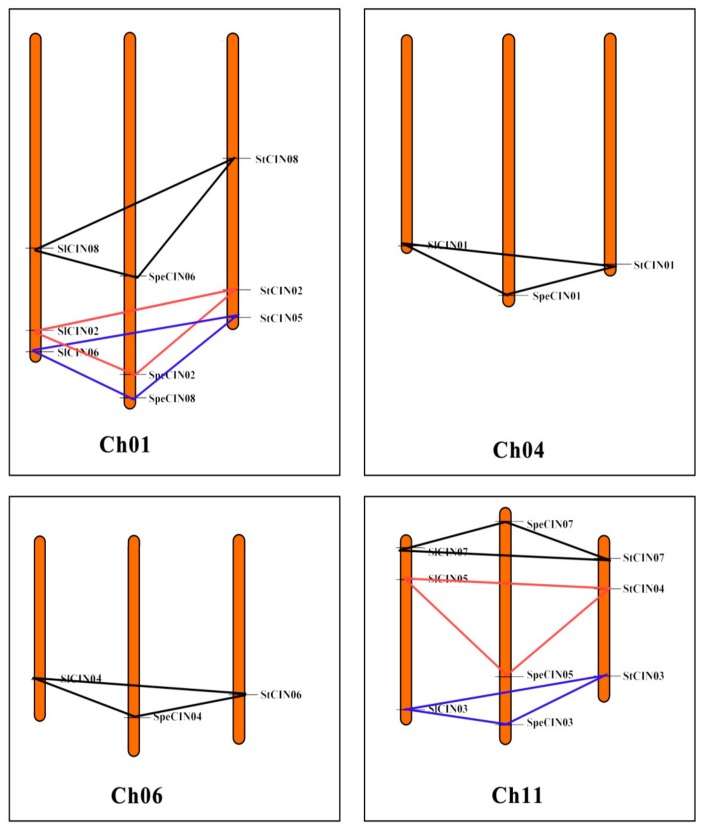
Collinearity relationship of *CIN* genes from *S. lycopersicum*, *S. pennellii*, and *S. tuberosum*. Collinear gene pairs tied with lines in different colors. Chromosome numbers shown at the bottom of each box.

It was previously reported that collinearity, a more specific form of synteny, was originally defined as a set of genes from two species located on the same chromosome that contained a conserved gene order during evolution [44,45]. When comparing the gene distribution between *S. lycopersicum*, *S. pennellii*, and *S. tuberosum*, a high degree of collinearity conservation was detected in these three species (Figure 6). Collinearity of the gene order was further illustrated when chromosome-by-chromosome comparison was made between the three *Solanum* species (Figure 7). 

A total of eight groups of collinear gene pairs were found, as shown in Figure 7. From them, multiple groups of *CIN* genes, forming orthologous genes to each other, are intimately clustered at one node of phylogenetic tree. These orthologous genes usually share a common ancestor. For example, *SlCIN01*, *SpeCIN01*, and *StCIN01* are orthologous genes of the β group; *SlCIN02*, *SpeCIN02*, and *StCIN02* belonging to the α group are also orthologous genes. Strong collinearity and abundance of orthologous gene pairs again support the idea that the CIN gene family was highly conservative in the process of evolution. Collinearity can be regarded as an ‘agent for the conservation or constraint of gene function’, and it can provide insight into the evolution of genomes [45,46,47]. Such strong collinearity in the CIN gene family can not only provide a clue to reveal conservation CINs, but also contributes to understanding the evolutionary and functional characterization of *CIN* genes. Particularly, the presence of massive collinearity indicated that CIN genes in the *Solanum* genome arose before the divergence of these different species. Additionally, no evidence was found for gene-duplication events (such as segmental and tandem duplication) in these 24 CIN genes. 

### 3.6. Cross-Species Transcript Profiling Reveals High Constitutive Expression of CIN Genes in *S. lycopersicum* and *S. tuberosum*

**Figure 8:**
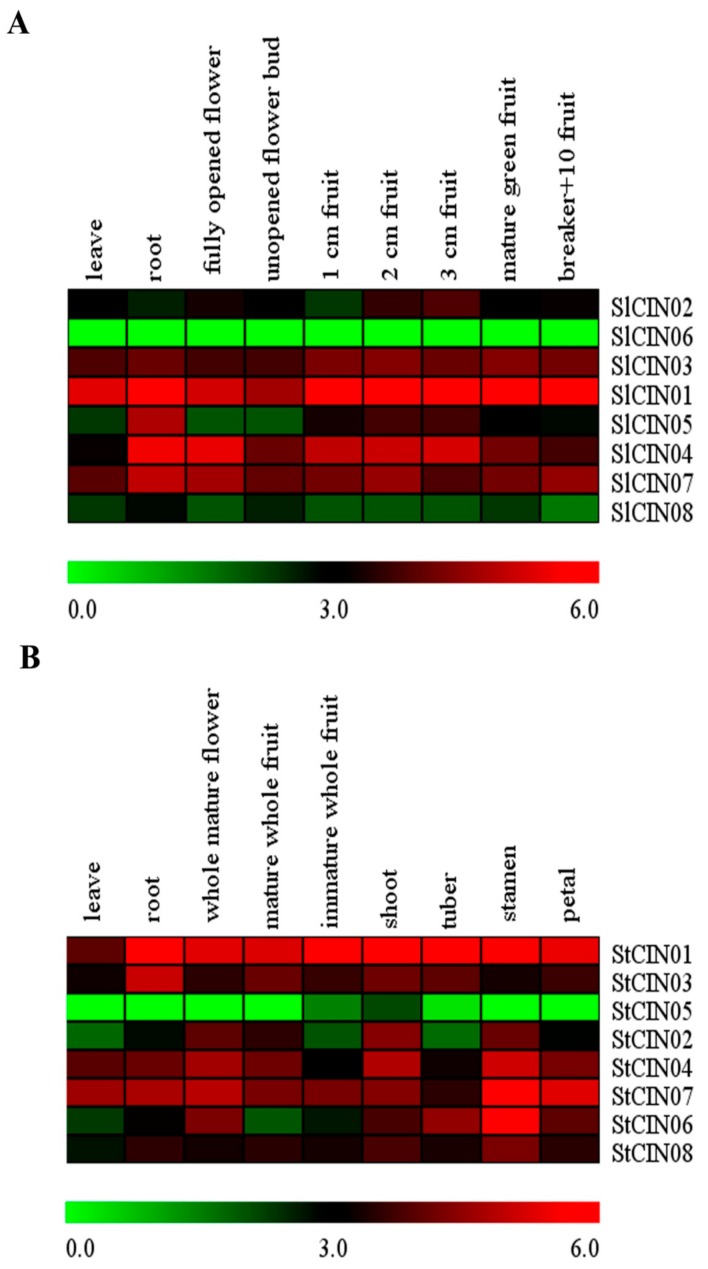
Heat map of expression profiles of CIN genes in various stages or tissue types of (**A**) *S. lycopersicum* and (**B**) *S. tuberosum*. (**A**) Tested tissue types were leaves, roots, fully opened flower, unopened flower bud, 1 cm fruit, 2 cm fruit, 3 cm fruit, mature green fruit, and breaker + 10 fruit in *S. lycopersicum*. (**B**) Tested tissue types were leaves, roots, whole mature flower, mature whole fruit, immature whole fruit, shoot, tuber, stamen, and petal in *S. tuberosum*. Fragments Per Kilobase Million mapped reads (FPKM) values were log_2_-transformed, and heat maps with hierarchical clustering exhibited using Mev 4.9.0 software.

Digital expression profiling is a particularly powerful and efficient method for large-scale gene-expression analysis [48,49,50,51,52,53,54,55], and it contributes to understanding gene functions. We therefore analyzed the expression profiles of the CIN gene family at different types of tissue and developmental stages from *S. lycopersicum* and *S. tuberosum* on the basis of *in silico* analysis (Figure 8). In *S. lycopersicum*, six out of eight paralog CIN genes were constitutively or broadly expressed in all analyzed samples (leaf, root, fully opened flower, unopened flower bud, 1–3 cm fruit, mature green fruit, and breaker + 10 fruit; Figure 8A). Correspondingly, a similar scenario was also observed in the CIN gene family of the potato genome (Figure 8B). It was previously reported that some genes that displayed broad expression generally exhibit higher conservation than that of those expressed in specific types of tissue for plant and mammal species [56,57]. Hence, the conservation of the CIN gene family was verified by constitutive expression profiling once again. 

Significantly, two CIN orthologs (*SlCIN06* and *StCIN05*) were unexpressed or hardly detected, and were phylogetically grouped into the same β subclade (Figure 1 and Figure 8). These findings hint that β CINs located on the cytosol might behave similarly in functional expression across species compared with the α group. By contrast, an expression level of SlCIN08 was not detected in the tomato genome, while the *StCIN08* potato gene was broadly expressed in all examined samples compared with *SlCIN08*. Both of them were clustered in the same subclade belonging to the α group. These observations also support the view mentioned above that CIN genes of the β group have higher stability and sequence similarity than those of the α group.

### 3.7. Expression Profiles of CIN Genes under Different Stress Conditions

**Figure 9:**
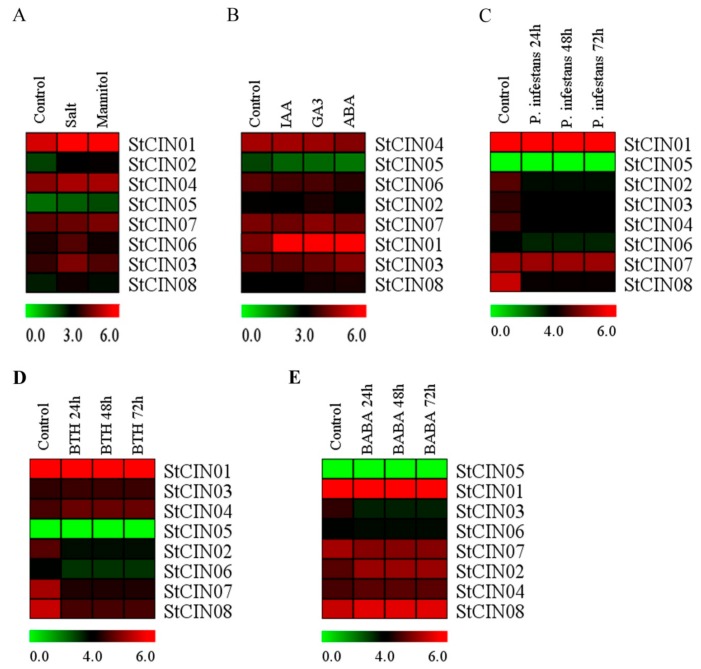
Expression profiles of potato CIN genes under various stress conditions. Changes of CIN gene transcription levels in response to (**A**) abiotic stresses, (**B**) different phytohormones, and (**C**–**E**) biotic stresses. FPKM values were log_2_-transformed, and heat maps with hierarchical clustering exhibited using Mev 4.9.0 software.

The gene expression patterns in response to abiotic, biotic stresses and phytohormones could provide information for potential gene function [58,59,60,61,62]. The expression profiles of potato CIN genes were analyzed in response to abiotic and biotic stresses, and phytohormones. Abiotic and biotic stresses included salt, mannitol and *Phytophthora infestans* (24/48/72 h), benzothiadiazole (BTH) (24/48/72 h), and β-aminobutyric acid (BABA) (24/48/72 h), respectively. Phytohormones were composed of indoleacetic acid (IAA), gibberellins (GA_3_), and abscisic acid (ABA). Analysis found that the transcription levels of almost all CIN genes were not detected in response to stresses and phytohormones (value changes less than twofold; Figure 9B). Only one gene (*StCIN01*) showed upregulation under phytohormones, and biotic stressing *P. infestans* induced the downregulation of the *StCIN08* gene (value changed less than twofold with P < 0.05) (Figure 9, Table S4). The *StCIN01* gene may have a specific function in hormone regulation. In addition, some genes were slightly up- or downregulated under treatments, like StCIN01 under abiotic stress (Figure 9A), *StCIN*02 under *P. infestans* (Figure 9C), *StCIN*01/02/07/08 under BTH (Figure 9D), and StCIN03 under BABA (Figure 9E).

### 3.8. Expression profiles of tomato CIN genes in response to phytohormones with qRT-PCR

**Figure 10:**
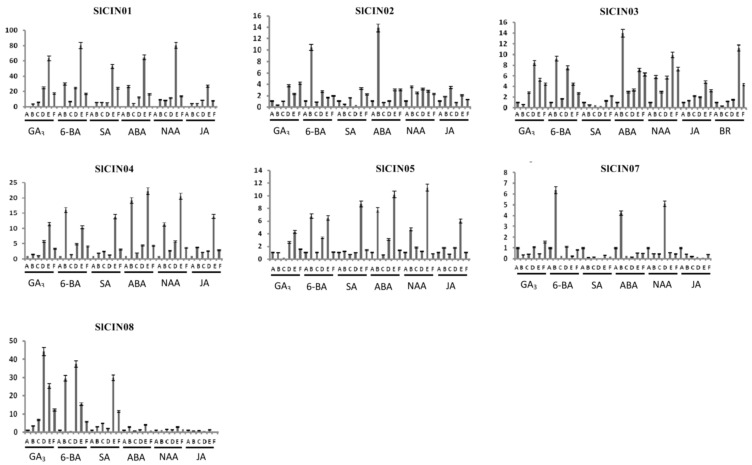
Expression profiles of tomato CIN genes in response to phytohormone treatments. Expression levels of these CINs under various phytohormone treatments were detected using qRT-PCR. Bar graphs indicate relative expression values of each CIN gene after phytohormone treatment. Leaf samples were collected at 0, 1.5, 3, 6, 12 and 24 h after treatment, respectively. Each time point of sample collection represented by A, B, C, D, E, and F, respectively. The mean of three biological replicates was selected for analysis.

qRT-PCR analysis was used to investigate expression levels of tomato CIN genes under different phytohormones (including GA_3_, 6-BA, SA, ABA, NAA and JA; Table S5). The *SlCIN06* gene was not selected for analysis due to invalid primers. Of the seven remaining genes, after treatment with different phytohormones, the *SlCIN01* gene was highly induced at 12 h, and transcription levels were steeply downregulated at 24 h (Figure 10). On the other hand, the *SlCIN08* gene was expressed at certain stages of GA_3_, 6-BA, and SA induction. The remaining genes (*SlCIN*02/03/04/05/07) were barely affected by phytohormones or were slightly induced at specific stages.

## 4. Conclusions

In the current study, the whole genomes of five *Solanum* species (*S. tuberosum*, *S. lycopersicum*, *S. pennellii*, *S. pimpinellifolium*, and *S. melongena*) were chosen to identify the CIN gene family. A total of 40 putative CIN genes were identified in the five plants, and each species contained a small CIN gene family with eight members. Subsequently, comparative analysis involving protein sequences, phylogenetic relationships, *cis*-acting elements, genome organization, and expression profile was performed at the CIN family level. On the basis of our results, an abundance of functional genomic evidence showed that the CIN gene family is evolutionarily and functionally conserved. In summary, our study not only provides a comprehensive understanding of the CIN gene family existing in *Solanum*, but also established a basis for further working out the function of CIN candidates in the whole Solanaceae family in the future.

## Figures and Tables

**Table 1:** List of 40 identified acid- and neutral/alkaline invertase (cytosolic invertase, CIN) members in *Solanum*.

Gene Name	Sequence ID	Chromosome Location	ORF Length (bp)	Intron Number	Protein Length (AA)	MW (KDA)
*StCIN01*	PGSC0003DMC400017548	ch04:70796961..70817020	1710	3	570	65.2
*StCIN02*	PGSC0003DMC400004942	ch01:78428855..78437323	1857	5	619	70.5
*StCIN03*	PGSC0003DMC400046100	ch11:39906240..39914966	1923	5	641	72.6
*StCIN04*	PGSC0003DMC400016331	ch11:13671979..13682038	1605	3	535	60.9
*StCIN05*	PGSC0003DMC400002890	ch01:87159564..87165074	1704	3	568	64.9
*StCIN06*	PGSC0003DMC400045302	ch06:46659148..46666904	1653	3	551	62.8
*StCIN07*	PGSC0003DMC400033886	ch11:5045582..5054168	1965	5	655	73.6
*StCIN08*	PGSC0003DMC400023170	ch01:37066183..37073554	2034	5	678	76.6
*SlCIN01*	Solyc04g081440.3.1	ch04:65414803..65419467	1710	3	570	65.2
*SlCIN02*	Solyc01g100810.3.1	ch01:90737614..90743352	1959	5	653	74.5
*SlCIN03*	Solyc11g067050.3.1	ch11:52804241..52807353	1923	5	672	76.2
*SlCIN04*	Solyc06g065210.4.1	ch06:40659646..40663785	1653	3	551	62.8
*SlCIN05*	Solyc11g020610.3.1	ch11:11747788..11752875	1656	3	552	62.6
*SlCIN06*	Solyc01g111100.4.1	ch01:97477472..97482570	1704	3	568	64.9
*SlCIN07*	Solyc11g007270.2.1	ch11:1652454..1656955	2010	6	670	75.2
*SlCIN08*	Solyc01g058010.3.1	ch01:64953319..64958618	1518	3	641	72.6
*SpeCIN01*	Sopen04g035050.1	ch04:76047959..76052613	1710	3	570	65.2
*SpeCIN02*	Sopen01g044310.1	ch01:101216445..101222621	1758	5	586	66.6
*SpeCIN03*	Sopen11g026760.1	ch11:62559567..62563895	1923	5	641	72.6
*SpeCIN04*	Sopen06g023710.1	ch06:51117483..51123347	1653	3	551	62.8
*SpeCIN05*	Sopen11g021020.1	ch11:48314836..48320989	1656	3	552	62.7
*SpeCIN06*	Sopen01g023510.1	ch01:71411679..71418653	1908	5	635	72.9
*SpeCIN07*	Sopen11g003160.1	ch11:1750266..1756318	1965	5	655	73.5
*SpeCIN08*	Sopen01g053160.1	ch01:108318819..108324218	1236	2	412	47.3
*SpiCIN01*	Sopim04g081440.0.1		1710	3	570	65.2
*SpiCIN02*	Sopim01g100810.0.1		1959	5	653	74.5
*SpiCIN03*	Sopim11g067050.0.1		1923	5	641	72.5
*SpiCIN04*	Sopim06g065210.0.1		1653	3	551	62.8
*SpiCIN05*	Sopim11g020610.0.1		1605	3	535	60.9
*SpiCIN06*	Sopim01g111100.0.1		1815	5	605	69.1
*SpiCIN07*	Sopim11g007270.0.1		1965	5	655	73.5
*SpiCIN08*	Sopim01g058010.0.1		1518	3	641	72.6
*SmCIN01*	Sme2.5_00505.1_g00017.1		1710	3	570	65.1
*SmCIN02*	Sme2.5_03894.1_g00007.1		1980	5	660	75.2
*SmCIN03*	Sme2.5_02773.1_g00004.1		1569	5	523	59.3
*SmCIN04*	Sme2.5_05261.1_g00002.1		1236	2	412	47.3
*SmCIN05*	Sme2.5_01346.1_g00001.1		1914	9	673	76.1
*SmCIN06*	Sme2.5_07789.1_g00007.1		1908	7	636	72.1
*SmCIN07*	Sme2.5_00977.1_g00009.1		1641	3	547	61.9
*SmCIN08*	Sme2.5_24471.1_g00001.1		810	6	294	34.1

MW, molecular weight; ORF, open reading frame; AA, amino acid.

## Supplementary Materials

The following are available online at https://www.mdpi.com/2218-273X/9/12/763/s1. Figure S1: Phylogenetic relationship of Solanum CINs with Arabidopsis. Phylogenetic tree of CIN proteins was constructed with the maximum-parsimony method using MEGA 7.0 software. Support values shown in each line in the phylogenetic tree. Putative CIN genes divided into two subfamilies (α and β) on the basis of their in silico prediction of subcellular localization; α subfamily further divided into α1 and α2. Table S1: Characterization of CIN gene family in α and β groups. Table S2: Multiple sequence alignment of CIN genes in *Solanum*. Table S3: Pairwise identity of CIN genes in *Solanum*. Table S4: Expression data of potato CIN genes under various stress conditions. Table S5: Primers used to detect expression of tomato invertase genes in this study.

## References

[1] Vargas W.A., Salerno G.L. (2010). The Cinderella story of sucrose hydrolysis: Alkaline/neutral invertases, from cyanobacteria to unforeseen roles in plant cytosol and organelles. Plant Sci..

[2] Avigad G., Dey P.M., Dey P.M., Harborne J.B. (1997). Carbohydrate metabolism: Storage carbohydrates. Plant Biochemistry.

[3] Ruan Y.L., Jin Y., Yang Y.J., Li G.J., Boyer J.S. (2010). Sugar input, metabolism, and signaling mediated by invertase: Roles in development, yield potential, and response to drought and heat. Mol. Plant..

[4] Koch K. (2004). Sucrose metabolism: Regulatory mechanisms and pivotal roles in sugar sensing and plant development. Curr. Opin. Plant Biol..

[5] Hummel M., Rahmani F., Smeekens S., Hanson J. (2009). Sucrose-mediated translational control. Ann. Bot..

[6] Ruan Y.L. (2014). Sucrose metabolism: Gateway to diverse carbon use and sugar signaling. Annu. Rev. Plant Biol..

[7] Smeekens S. (2000). Sugar-induced signal transduction in plants. Annu. Rev.Plant Physiol. Plant Mol. Biol..

[8] Rolland F., Moore B., Sheen J. (2002). Sugar sensing and signaling in plants. Plant Cell..

[9] Coleman H.D., Yan J., Mansfield S.D. (2009). Sucrose synthase affects carbon partitioning to increase cellulose production and altered cell wall ultrastructure. Proc. Natl. Acad. Sci. USA.

[10] Roitsch T., Gonzàlez M.C. (2004). Function and regulation of plant invertases: Sweet sensations. Trends Plant Sci..

[11] Wang L., Ruan Y.L. (2016). Critical roles of vacuolar invertase in floral organ development and male and female fertilities are revealed through characterization of GhVIN1-RNAi cotton plants. Plant Physiol..

[12] Barratt D.H., Derbyshire P., Findlay K., Pike M., Wellner N., Lunn J., Feil R., Simpson C., Maule A.J., Smith A.M. (2009). Normal growth of Arabidopsis requires cytosolic invertase but not sucrose synthase. Proc. Natl. Acad. Sci. USA.

[13] Sturm A. (1999). Invertases Primary structures, functions, and roles in plant development and sucrose partitioning. Plant Physiol..

[14] Salerno G.L., Curatti L. (2003). Origin of sucrose metabolism in higher plants: When, how and why?. Trends Plant Sci..

[15] Vargas W., Cumino A., Salerno G.L. (2003). Cyanobacterial alkaline/neutral invertases: Origin of sucrose hydrolysis in the plant cytosol?. Planta.

[16] Goetz M., Guivarćh A., Hirsche J., Bauerfeind M.A., González M.C., Hyun T.K., Eom S.H., Chriqui D., Engelke T., Großkinsky D.K. (2017). Metabolic control of tobacco pollination by sugars and invertases. Plant Physiol..

[17] Swarbrick P.J., Lefert P.S., Scholes J.D. (2006). Metabolic consequences of susceptibility and resistance (race-specific and broad-spectrum) in barley leaves challenged with powdery mildew. Plant Cell Environ..

[18] Liu Y.H., Ruan Y.L. (2016). Cell wall invertase promotes fruit set under heat stress by suppressing ROS-independent cell death. Plant Physiol..

[19] Qi X., Wu Z., Li J., Mo X., Wu S., Chu J., Wu P. (2007). AtCYT-INV1, a neutral invertase, is involved in osmotic stress-induced inhibition on lateral root growth in *Arabidopsis*. Plant Mol. Biol..

[20] Welham T., Pike J., Horst I., Flemetakis E., Katinakis P., Kaneko T., Sato S., Tabata S., Perry J., Parniske M.A. (2009). A cytosolic invertase is required for normal growth and cell development in the model legume, Lotus Japonicus. J. Exp. Bot..

[21] Gao J., van Kleeff P.J., Oecking C., Li K.W., Erban A., Kopka J., Hincha D.K., de Boer A.H. (2014). Light modulated activity of root alkaline/neutral invertase involves the interaction with 14-3-3 proteins. Plant J..

[22] Xie J., Cai K., Hu H.X., Jiang Y.L., Yang F., Hu P.F., Cao D.D., Li W.F., Chen Y., Zhou C.Z. (2016). Structural analysis of the catalytic mechanism and substrate specificity of Anabaena alkaline invertase InvA reveals a novel glucosidase. J. Biol. Chem..

[23] Xie J., Hu H.X., Cai K., Yang F., Jiang Y.L., Chen Y., Zhou C.Z. (2018). Structural and enzymatic analyses of Anabaena heterocyst-specific alkaline invertase InvB. FEBS Lett..

[24] Wan H.J., Wu L., Yang Y., Zhou G., Ruan Y.L. (2018). Evolution of sucrose metabolism: The dichotomy of invertases and beyond. Trends Plant Sci..

[25] Nonis A., Ruperti B., Pierasco A., Canaguier A., Adam-Blondon A.F., Gaspero G., Vizzotto G. (2008). Neutral invertases in grapevine and comparative analysis with *Arabidopsis*, poplar and rice. Planta.

[26] Shen L.B., Yao Y., He H., Qin Y.L., Liu Z.J., Liu W.X., Qi Z.Q., Yang L.J., Cao Z.M., Yang Y. (2018). Genome-wide identification, expression, and functional analysis of the alkaline/neutral invertase gene family in pepper. Int. J. Mol. Sci..

[27] Yao S.G., Kodama R., Wang H., Ichii M., Taketa S., Yoshida H. (2009). Analysis of the rice SHORT-ROOT5 gene revealed functional diversification of plant neutral/alkaline invertase family. Plant Sci..

[28] Liu S., Lan J., Zhou B., Qin Y., Zhou Y., Xiao X., Yang J., Gou J., Qi J., Huang Y. (2015). HbNIN2, a cytosolic alkaline/neutral-invertase, is responsible for sucrose catabolism in rubber-producing laticifers of *Hevea brasiliensis* (para rubber tree). New Phytol..

[29] Hall T.A. (1999). BioEdit: A user-friendly biological sequence alignment editor and analysis program for Windows 95/98/NT. Nucl. Acids Symp. Ser..

[30] Larkin M.A., Blackshields G., Brown N.P., Chenna R., McGettigan P.A., McWilliam H., Valentin F., Wallace I.M., Wilm A., Lopez R. (2007). Clustal W and Clustal X version 2.0. Bioinformatics.

[31] Kumar S., Stecher G., Tamura K. (2016). MEGA7: Molecular evolutionary genetics analysis version 7.0 for bigger datasets. Mol. Biol. Evol..

[32] Chen C., Chen H., He Y., Xia R. (2018). TBtools, a Toolkit for Biologists integrating various biological data handling tools with a user-friendly interface. bioRxiv.

[33] Liu R.H., Meng J.L. (2003). MapDraw: A microsoft excel macro for drawing genetic linkage maps based on given genetic linkage data. Hereditas (Beijing).

[34] Gu Z., Cavalcanti A., Chen F.C., Bouman P., Li W.H. (2002). Extent of gene duplication in the genomes of Drosophila, nematode, and yeast. Mol. Biol. Evol..

[35] Yang S., Zhang X., Yue J.X., Tian D., Chen J.Q. (2008). Recent duplications dominate NBS-encoding gene expansion in two woody species. Mol. Genet. Genomics.

[36] Howe E., Holton K., Nair S., Schlauch D., Sinha R., Quackenbush J., Ochs M., Casagrande J., Davuluri R. (2010). MeV: MultiExperiment viewer. Biomedical Informatics for Cancer Research.

[37] Livak K.J., Schmittgen T.D. (2001). Analysis of relative gene expression data using real-time quantitative PCR and the 2 ^-△△^ct method. Methods.

[38] Tunca D., Bilge K. (2013). Automatic identification of highly conserved family regions and relationships in genome wide datasets including remote protein sequences. PLoS ONE.

[39] Murayama S., Handa H. (2007). Genes for alkaline/neutral invertase in rice: alkaline/neutral invertases are located in plant mitochondria and also in plastids. Planta.

[40] Hirakawa H., Shirasawa K., Miyatake K., Nunome T., Negoro S., Ohyama A., Yamaguchi H., Sato S., Isobe S., Tabata S. (2014). Draft genome sequence of eggplant (*Solanum melongena* L): The representative *Solanum* species indigenous to the old world. DNA Res..

[41] Hou Y.B., Lin S.J. (2009). Distinct gene number-genome size relationships for eukaryotes and non-eukaryotes: Gene content estimation for dinoflagellate genomes. PLoS ONE.

[42] Nakano T., Suzuki K., Fujimura T., Shinshi H. (2006). Genome-wide analysis of the ERF gene family in *Arabidopsis* and rice. Plant Physiol..

[43] Ji X., Van E.W., Van L.A., Cheng S., Bennett J. (2005). Structure, evolution, and expression of the two invertase gene families of rice. J. Mol. Evol..

[44] Zhao T., Rens H., Suzanne D.B., Gerco C.A., Burg H.A., Schranz M.E. (2017). Phylogenomic synteny network analysis of MADS-Box transcription factor genes reveals lineage-specific transpositions, ancient tandem duplications, and deep positional conservation. Plant Cell.

[45] Wang Y.P., Tang H.B., Jeremy D.D., Tan X., Li J.P., Wang X.Y., Lee T., Jin H.Z., Barry M., Guo H. (2012). MCScanX: A toolkit for detection and evolutionary analysis of gene synteny and collinearity. Nucleic Acids Res..

[46] Dewey C.N. (2011). Positional orthology: Putting genomic evolutionary relationships into context. Brief Bioinform..

[47] Lv J., Havlak P., Putnam N.H. (2011). Constraints on genes shape long-term conservation of 887 macro-synteny in metazoan genomes. BMC Bioinform..

[48] Wang Z., Gerstein M., Snyder M. (2009). RNA-Seq: A revolutionary tool for transcriptomics. Nat. Rev. Genet..

[49] Lu T., Zhang G., Sun L., Wang J., Hao F. (2017). Genome-wide identification of CBL family and expression analysis of CBLs in response to potassium deficiency in cotton. Peer J..

[50] Chen M.Y., Li K., Li H.P., Song C.P., Miao Y.C. (2017). The Glutathione peroxidase gene family in *Gossypium hirsutum*: Genome-wide identification, classification, gene expression and functional analysis. Sci. Rep..

[51] Zhang G., Lu T., Miao W., Sun L., Tian M., Wang J., Hao F. (2017). Genome-wide identification of ABA receptor PYL family and expression analysis of PYLs in response to ABA and osmotic stress in *Gossypium*. Peer J..

[52] Sun Q., Wang G.H., Zhang X., Zhang X.R., Qiao P., Long L., Yuan Y.L., Cai Y.F. (2017). Genome-wide identification of the TIFY gene family in three cultivated *Gossypium* species and the expression of JAZ genes. Sci. Rep..

[53] Liu L.Y., Li N., Yao C.P., Meng S.S., Song C.P. (2013). Functional analysis of the ABA-responsive protein family in ABA and stress signal transduction in Arabidopsis. Chin. Sci. Bull..

[54] Guo Y.W., Guo H.L., Li X., Huang L.L., Zhang B.N., Pang X.B., Liu B.Y., Ma L.Q., Wang H. (2013). Two type III polyketide synthases from *Polygonum cuspidatum*: Gene structure, evolutionary route and metabolites. Plant Biotech. Rep..

[55] Xia X., Zhang H.M., Offler C.E., Patrick J.W. (2019). Enzymes contributing to the hydrogen peroxide signal dynamics that regulate wall labyrinth formation in transfer cells. J. Exp. Bot..

[56] Akashi H. (2001). Gene expression and molecular evolution. Curr. Opin. Genet. Dev..

[57] Wright S.I., Yau C.B., Looseley M., Meyers B.C. (2004). Effects of gene expression on molecular evolution in *Arabidopsis thaliana* and *Arabidopsis lyrata*. Mol. Biol. Evol..

[58] Duret L., Mouchiroud D. (2000). Determinants of substitution rates in mammalian genes, expression pattern affects selection intensity but not mutation rate. Mol. Biol. Evol..

[59] Hao F., Zhao S., Dong H., Zhang H., Sun L., Miao C. (2010). Nia1 and Nia2 are involved in exogenous salicylic acid-induced nitric oxide generation and stomatal closure in *Arabidopsis*. J. Integr. Plant Biol..

[60] Xu L.H., Wang W.Y., Guo J.J., Qin J., Shi D.Q., Li Y.L., Xu J. (2014). Zinc improves salt tolerance by increasing reactive oxygen species scavenging and reducing Na+ accumulation in wheat seedlings. Biol. Plant..

[61] Wang P.T., Liu H., Hua H.J., Wang L., Song C.P. (2011). A vacuole localized β-glucosidase contributes to drought tolerance in Arabidopsis. Chinese Sci. Bull..

[62] Qi J., Song C.P., Wang B., Zhou J., Kangasjärvi J., Zhu J.K., Gong Z. (2018). Reactive oxygen species signaling and stomatal movement in plant responses to drought stress and pathogen attack. J. Integr. Plant Biol..

