# Supplementary material for: Evolutionary Conservation and Expression Patterns of Neutral/Alkaline Invertases in Solanum
Source: Biomolecules. 2019 Nov 21;9(12):763. doi: 10.3390/biom9120763 (PMC6995568; doi:10.3390/biom9120763)
Supplement: Supplementary file 1 [file biomolecules-09-00763-s001.zip › Supplemental Table S5.docx]

Supplemental Table S5 Primers used to detect expression of tomato invertase genes in this study

| Gene | Primer name | Forward primer (5' to 3') | Reverse primer (5' to 3') |
| --- | --- | --- | --- |
| Solyc01g100810 | SlCIN1 | CTACCCTGAACAAATCCCTC | GGCATAAGACCCACAAGATC |
| Solyc04g081440 | SlCIN2 | TAGTTGGAGAAATGCCGCTG | GTAAGGAGCCACAGAAGAAC |
| Solyc06g065210 | SlCIN3 | CACGAATGGCGGATAGTAAC | ACCAATGTATCTGCCGAGCT |
| Solyc11g007270 | SlCIN4 | GTCTTGCTGATGGCTTTGAT | TCCTCCTTTACTTGGCATCC |
| Solyc11g020610 | SlCIN5 | TATTGTCCTCCCTTGCCACT | ATTTGTGGTCGTCCCGTCTT |
| Solyc11g067050 | SlCIN7 | TGGCTGGTGGAATGGATGC | CACCACCAGTAATAATGCGC |
| Solyc01g058010 | SlCIN8 | CGTGGACTGCTATAACCCTGG | ATAACCCACCACAACCCAGAA |
| U97257 | GAPDH | GGCTGCAATCAAGGAGGAA | AAATCAATCACACGGGAACTG |
