# Supplementary material for: Evolutionary Conservation and Expression Patterns of Neutral/Alkaline Invertases in Solanum
Source: Biomolecules. 2019 Nov 21;9(12):763. doi: 10.3390/biom9120763 (PMC6995568; doi:10.3390/biom9120763)
Supplement: Supplementary file 1 [file biomolecules-09-00763-s001.zip › Supplementary Table S2.docx]

**10 20 30 40 50 60 70 80 90 100**

**....|....| ....|....| ....|....| ....|....| ....|....| ....|....| ....|....| ....|....| ....|....| ....|....|**

---------- ---------- ---------- ---------- ---------- ---------- ---------- ---------- ---------- ---------- **SlCIN01**

---------- ---------- ---------- ---------- ---------- ---------- ---------- ---------- ---------- ---------- **SpiCIN01**

---------- ---------- ---------- ---------- ---------- ---------- ---------- ---------- ---------- ---------- **SpeCIN01**

---------- ---------- ---------- ---------- ---------- ---------- ---------- ---------- ---------- ---------- **StCIN01**

---------- ---------- ---------- ---------- ---------- ---------- ---------- ---------- ---------- ---------- **SmCIN01**

---------- ---------- ---------- ---------- ---------- ---------- ---------- ---------- ---------- ---------- **SmCIN08**

---------- ---------- ---------- ---------- ---------- ---------- ---------- ---------- ---------- ---------- **SlCIN04**

---------- ---------- ---------- ---------- ---------- ---------- ---------- ---------- ---------- ---------- **SpiCIN04**

---------- ---------- ---------- ---------- ---------- ---------- ---------- ---------- ---------- ---------- **SpeCIN04**

---------- ---------- ---------- ---------- ---------- ---------- ---------- ---------- ---------- ---------- **StCIN06**

---------- ---------- ---------- ---------- ---------- ---------- ---------- ---------- ---------- ---------- **SlCIN05**

---------- ---------- ---------- ---------- ---------- ---------- ---------- ---------- ---------- ---------- **SpeCIN05**

---------- ---------- ---------- ---------- ---------- ---------- ---------- ---------- ---------- ---------- **SpiCIN05**

---------- ---------- ---------- ---------- ---------- ---------- ---------- ---------- ---------- ---------- **StCIN04**

---------- ---------- ---------- ---------- ---------- ---------- ---------- ---------- ---------- ---------- **SmCIN03**

---------- ---------- ---------- ---------- ---------- ---------- ---------- ---------- ---------- ---------- **SlCIN06**

---------- ---------- ---------- ---------- ---------- ---------- -----MQTRS STNNDPKSIP PFIYFGRRMS SELICKSIEA **SpiCIN06**

---------- ---------- ---------- ---------- ---------- ---------- ---------- ---------- ---------- ---------- **StCIN05**

---------- ---------- ---------- ---------- ---------- ---------- ---------- ---------- ---------- ---------- **SpeCIN08**

---------- ---------- ---------- ---------- ---------- ---------- ---------- ---------- ---------- ---------- **SmCIN04**

---------- -----MNTSS CISISTMKPC CRILSSCKGS SFIG----KC NHFINDNLSN PHCKLDDIHT VSDYATRAIG ---IIGSNRS FFCGS-DSNW **SlCIN08**

---------- -----MNTSS CISISTMKPC CRILSSCKGS SFIG----KC NHFINDNLSN PHCKLDDIHT VSDYATRAIG ---IIGSNRS FFCGS-DSNW **SpiCIN08**

---------- -----MNTSS CIGISTMKPC CRILSSCKGS SFIGYSFGKC NHLINDNLSN PHCKLDDIHR VSNYANRVIG ---VIGSNRS VFCGS-DSNW **StCIN08**

---------- ---------- ---------- ---------- ---------- ---MNDNLSN PHCKLDDIHT VSDYATRVIG ---IIGSNRS VFCGS-DSNW **SpeCIN06**

---------- ---------- ------MKPC CRILRSCKGS STFG----KC NHLISDNVSK PHCRSDDIHR VSDYANRAIE ---VIGLNRS VFCGS-DLNW **SmCIN02**

---------- --------MK SINLITMTPC CRILIPCRSN SFLGLPFKKT HN-LS----- ---NFRQKCD FYSYPSRILG NGRIINRTQK LFCVMRNSSC **SlCIN02**

---------- --------MK SINLITMTPC CRILIPCRSN SFLGLPFKKT HN-LS----- ---NFRQKCD FYSYPSRILG NGRIINRTQK LFCVMRNSSC **SpiCIN02**

---------- ---------- ---------- ---------- ---------- ---------- ---------- ---------- ---------- ----MRNSSC **SpeCIN02**

---------- ---------- ---------- ---------- ---------- ---MS----- ---NFRQKCD FHSYPSRILG NGRIINRTQK LFCVVRNSSC **StCIN02**

---------- ---------- ------MAPC CRILIPCRSN SFLGLPFKKT HNSFSKNSSN FRLNFRQKCD FHGYPSRSLG TGRISNRTQK LFCVVRNSSC **SmCIN07**

----MLFHVN VLIKSRNYYN SCKAEGIINF RYLLTMGASE AALQLLSGEL SCQVRT--SS ILAKSNSLLC YERCFKARNY GDWR-YKQIN SIKKLQDCSS **SlCIN03**

---------- ---------- ---------- -----MGASE AALQLLSGEL SCQVRT--SS ILAKSNSLLC YERCFKARNY GDWR-YKQIN SIKKLQDCSS **SpiCIN03**

---------- ---------- ---------- -----MGASE AALQLLSSEL SCQVRT--SS ILVKSNSLLC YERCFKARNY GDWR-YKQIN GIKKLQDCSS **SpeCIN03**

---------- ---------- ---------- -----MGASE AALQLLSGTL SFQVRT--SS ILAKSNSLLC YERCFKARNC GDWR-YKQIK GLKKLQDCSS **StCIN03**

MSIGNGVSCY AVLVLVNYYS SCKAEGIINF RYLLTMGASE ASLQLLSGEI SCQVRT--SS ILAKSNSLLC YEGCAKTRGY EDWR-YKQIK GLKGLQNCSR **SmCIN05**

---------- ---------- ---------- -----MATSE AFLQVLGGSL PSLFGS--DD SFRKLGSSHT SRSFIRIRKK RGPICVNFLN CSHISYRAIR **SlCIN07**

---------- ---------- ---------- -----MATSE AFLQVLGGSL PSLFGS--DD SFRKLGSSHT SRSFIRIRKK RGPICVNFLN CSHISYRAIR **SpiCIN07**

---------- ---------- ---------- -----MATSE AFLQVIGGSL PSLFGS--DD SFRKLGSSHT SRSFIRIRKK RGSMCVNFLN CSNISYRAIR **SpeCIN07**

---------- ---------- ---------- -----MATSE AVLQVLGGSL PSLFGS--DN SFRKLGSSHT SRSFIRIRKK RGSKCVNFLN CSNISYRAIR **StCIN07**

---------- ---------- ---------- ---------- -MIEFYS--- -KLYTE--PE SWR---HSFE FRGCPTISAE EN----EWLQ RS-----STE **SmCIN06**

**Clustal Conse**

**110 120 130 140 150 160 170 180 190 200**

**....|....| ....|....| ....|....| ....|....| ....|....| ....|....| ....|....| ....|....| ....|....| ....|....|**

----MPSPVD VSQNGNARHA EAAPSLFEIE EDLARLLERP RQVNIERKRS FDERSFSEMS MTHSPPRQVY KNSENSSRVF DNMVGVYSPG RWSGIHTPRS **SlCIN01**

----MPSPVD VSQNGNARHA EAAPSLFEIE EDLARLLERP RQVNIERKRS FDERSFSEMS MTHSPPRQVY KNSENSSRVF DNMVGVYSPG RWSGIHTPRS **SpiCIN01**

----MPSPVD VSQNGNARHA EAAPSLFEIE EDLARLLERP RQVNIERKRS FDERSFSEMS MTHSPPRQVY KNSENSSRVF DNMVGVYSPG RWSGIHTPRS **SpeCIN01**

----MPSPVD VSQNGNARQA EAAPSLFEIE EDLARLLERP RQVNIERKRS FDERSFSEMS MTHSPPRQVY KNSENSSRVF DNMVGVYSPG RWSGIHTPRS **StCIN01**

----MPSPVD VSQNGSARHA EAAPSLFEIE EDLARLLERP RQVNIERKRS FDERSFSEMS MTHSPPRQVY RNSDNSSRVF DNMVGVYSPG RWSGIQTPRS **SmCIN01**

---------- ---------- ---------- ---------- ---------- ---------- ---------- ---------- ---------- ---------- **SmCIN08**

---------- -MEETGLRNV GSNCSISEID DYDLSKLLNK PRLNIERKRS FDERSLSELS IGLS------ -------RGL DHYESAS-PG QSVLDTPVSS **SlCIN04**

---------- -MEETGLRNV GSNCSISEID DYDLSKLLNK PRLNIERKRS FDERSLSELS IGLS------ -------RGL DHYESAS-PG QSVLDTPVSS **SpiCIN04**

---------- -MEETGLRNV GSNCSISEID DYDLSKLLNK PRINIERKRS FDERSLSELS VGLS------ -------RGL DHYESAS-PG QSVLDTPVSS **SpeCIN04**

---------- -MEETGLRNV GSNCSISEID DYDLSKLLNK PRLNIERKRS FDERSLSELS IGLS------ -------RGL DHYESAS-PG RSVLDTPVSS **StCIN06**

---------- -MEGSGLKNV SSHCSISEMD DFDLSKLLDK PRINIERQRS FDERSLSELS IGLS------ -------RGL DNYENAYSPG RSGLDTPASS **SlCIN05**

---------- -MEGSGLKNV SSHCSISEMD DFDLSKLLDK PRINIERQRS FDERSLSELS IGLS------ -------RGL DNYENAYSPG RSGLDTPASS **SpeCIN05**

---------- ---------- --------MD DFDLSKLLDK PRINIERQRS FDERSLSELS IGLS------ -------RGL DNYENAYSPG RSGLDTPASS **SpiCIN05**

---------- ---------- --------MD DFDLSKLLDK PRINIERQRS FDERSLSELS IGLS------ -------RGL DNYENTYSPG RSGLDTPASS **StCIN04**

---------- ---------- ---------- ---------- ---------- ---------- ---------- ---------- ---------- ---------- **SmCIN03**

--MSTHSGDV SNNDASIRNI DSCSTVTELD DIDFSRLPRP RNLNIERQGS YDEKSLTETQ LGFSP--HPP SRAENFFRAL EHFDCIFSPS KRSEFTTPRS **SlCIN06**

SRMSTHSGDV SNNDASIRNI DSCSTVTELD DIDFSRLPRP RNLNIERQGS YDEKSLTETQ LGFSP--HPP SRAENFFRAL EHFDCIFSPS KRSEFTTPRS **SpiCIN06**

--MSTHSGDV SNNDSSIRNI DSCSSVTELE DIDFSRLPRP RNLNIERQGS YDEKSLTETQ LGFSP--HPP SRAENFFRAL EHFDCIFSPS KRSEFTTPRS **StCIN05**

---------- ---------- ---------- ---------- ---------- ---------- ---------- ---------- ---------- ---------- **SpeCIN08**

---------- ---------- ---------- ---------- ---------- ---------- ---------- ---------- ---------- ---------- **SmCIN04**

RH-------- ---FRLNKET RCYSVDANVA SDGRNFSTSI EA-QVN-EKR FNKFYIQGCL NVK-PLVIDR IESGKDVAKV EEEIRTDINN GS-GVYVKHP **SlCIN08**

RH-------- ---FRLNKET RCYSVDANVA SDGRNFSTSI EA-QVN-EKR FNKFYIQGCL NVK-PLVIDR IESGKDVAKV EEEIRTDINN GS-GVYVKHP **SpiCIN08**

RHARILLG-- ---FRLNKET RCYCVNANAA SDVRNHSTSI EA-QVN-EKI FDKFYIHGGL NVK-PLVIDR KESGKDVAKV EK-VRTDVND GS-GVNVKHP **StCIN08**

RH-------- ---FRLNKET RCYSVDANVA SDVRNFSTSI EA-QVN-EKR FNKFYIQGCL NVK-PLVIDR IESGKDVAKV EEEIRTDINN GS-GVNVKHP **SpeCIN06**

RQSRVLFG-- ---FRLNKET RCHSVNANVA SGVRNHSTSI EA-QVD-DKS FDKFYIQGGL NMK-PLIIER IESGKDVAKV EQ-VRTVIND GS-TVN---P **SmCIN02**

GQSRVFSRNF NGINPMGTSK RGFRVIASVA SDFRNHSTSI EKTRVNNDKN FERIYVQGGF NAKKPLG--- ------LENA DLDEHAATGQ HEK------V **SlCIN02**

GQSRVFSRNF NGINPMGTSK RGFRVIASVA SDFRNHSTSI EKTRVNNDKN FERIYVQGGF NAKKPLG--- ------LENA DLDEHAATGQ HEK------V **SpiCIN02**

GQSRVFSRNC NGINPIGASK RGFRVIASVA SDFRNHSTSI EKTRVNNDKN FERIYVQGGF NAKKPLG--- ------LENA DLDEHAATGQ HEK------V **SpeCIN02**

GQSRVFSRNC NGINPIGASK RGFHVIASVA SDFRNHSTSV EKTRVNNDKN FERIYVQGGL NAKKPLG--- ------LENA DLDEHAATGQ HEK------V **StCIN02**

VQSRVFSRNC NGVNPIAASK RSFRVIANVA SDFRNYSTSI EKNRVN-DKN FERIYVQGGL GAKKPLVVED ADLNEHMENA DINEHGATGR REK------V **SmCIN07**

LHAFHGLHSV FCGEKLLSQS NLLICNCQQP ERVSETIIKG GNG------- KSMHTVSPKI PNLAPDE--- -----QNMKQ ENGARPFSEG ---FKTAASV **SlCIN03**

LHAFHGLHSV FCGEKLLSQS NLLICNCQQP ERVSETIIKG GNG------- KSMHTVSPKI PNLAPDE--- -----QNMKQ ENGARPFSEG ---FKTAASV **SpiCIN03**

LHAFHGLHSV FRGEKLHSQS NLLICNYQQP ERVSETIIKG GNG------- KSMHTVSPKI PNLAPDE--- -----QNMKQ ENGARPFSEG ---FKTAASV **SpeCIN03**

LHAFRGLHSV FRGEKIYNRS NLSICNCQQP ERVSETIIKG GNG------- KSMHTVPPKI PNLTPDE--- -----QNMKQ ENGARPFSEG ---FKTAASV **StCIN03**

LHAFRGLHSV FRGEKLHSRS NLLICNCQQP KRISETIING ENG------- KSMHTVSPKI PNHTPDE--- -----QNMKQ ENAARPFSEG ---FKTAAAV **SmCIN05**

VDCFQSTRQC VHGDIGHSNL RSVNCKCQQA DSASSFASEK GNGSWTIDND QSFDTVHGNT PSVMQFE--- -----TVREL KVGEENFQSN GSLPPNGLVE **SlCIN07**

VDCFQSTRQC VHGDIGHSNL RSVNCKCQQA DSASSFASEK GNGSWTIDND QSFDTVHGNT PSVMQFE--- -----TVREL KVGEENFQSN GSLPPNGLVE **SpiCIN07**

VDCFQSTRQS VHGDIGHSNL RSVNCKCQQA DSASSFASEK GNGSWTIDND QSFDTVHGNT PSVMQFE--- -----TVREL KVGEENFQSN GSLPPNGLVE **SpeCIN07**

VDCFQSIRQS VHGDITHSYL RSVNCKCQQA DSASSFASEK GNGSWISDND QSFDTVLGNT PSVMQFE--- -----TVREL KVGEEDFQSN GSLRPNVSAE **StCIN07**

EEILQTIKQN ------HSDL RSVNCKCQQT DSSSSFVSDK GNGSWIIDNG QSIDTIHGNT PSVLQFE--- -----TSKEL KVGEDDFQSN GSSPPNGSVE **SmCIN06**

**Clustal Conse**

**210 220 230 240 250 260 270 280 290 300**

**....|....| ....|....| ....|....| ....|....| ....|....| ....|....| ....|....| ....|....| ....|....| ....|....|**

T-FGYEP--- --------HP IIGEAWEALR RSIVNFRDQP VGTIAAIDNS ---AEELNYD QVFVRDFVPS ALAFLMNGEP DIVKNFLLKT LRLQS----- **SlCIN01**

T-FGYEP--- --------HP IIGEAWEALR RSIVNFRDQP VGTIAAIDNS ---AEELNYD QVFVRDFVPS ALAFLMNGEP DIVKNFLLKT LRLQS----- **SpiCIN01**

T-FGYEP--- --------HP IIGEAWEALR RSIVNFRDQP VGTIAAIDNS ---AEELNYD QVFVRDFVPS ALAFLMNGEP DIVKNFLLKT LRLQS----- **SpeCIN01**

T-FGYEP--- --------HP IIGEAWEALR RSIVNFRDQP VGTIAAIDNS ---AEELNYD QVFVRDFVPS ALAFLMNGEP DIVKNFLLKT LRLQS----- **StCIN01**

T-FGYEP--- --------HP IIGEAWEALR RSMVNFRGQP VGTIAAIDNS ---AEELNYD QVFVRDFVPS ALAFLMNGEP DIVKNFLLKT LRLQS----- **SmCIN01**

---------- ---------- ---------- ---------- ---------- ---------- ---------- ---------- ---------- ---------- **SmCIN08**

ARNSFEP--- --------HP MVAEAWDALR RTMVHFRGQP VGTIAAIDHA S--EEVLNYD QVFVRDFVPS ALAFLMNGEP DIVKNFLLKT LQLQG----- **SlCIN04**

ARNSFEP--- --------HP MVAEAWDALR RTMVHFRGQP VGTIAAIDHA S--EEVLNYD QVFVRDFVPS ALAFLMNGEP DIVKNFLLKT LQLQG----- **SpiCIN04**

ARNSFEP--- --------HP MVAEAWDALR RTMVHFRGQP VGTIAAIDHA S--EEVLNYD QVFVRDFVPS ALAFLMNGEP DIVKNFLLKT LQLQG----- **SpeCIN04**

ARNSFEP--- --------HP MVAEAWDALR RTMVHFRGQP VGTIAAIDHA S--EEVLNYD QVFVRDFVPS ALAFLMNGEP DIVKNFLLKT LQLQG----- **StCIN06**

ARNSFEP--- --------HP MVAEAWDALR RSMVHFRGQP VGTIAAVDHA A--EEVLNYD QVFVRDFVPS ALAFLMNGEP DIVKNFLLKT LQLQG----- **SlCIN05**

ARNSFEP--- --------HP MVAEAWDALR RSMVHFRGQP VGTIAAVDHA A--EEVLNYD QVFVRDFVPS ALAFLMNGEP DIVKNFLLKT LQLQG----- **SpeCIN05**

ARNSFEP--- --------HP MVAEAWDALR RSMVHFRGQP VGTIAAVDHA A--EEVLNYD QVFVRDFVPS ALAFLMNGEP DIVKNFLLKT LQLQG----- **SpiCIN05**

ARNSFEP--- --------HP MVAEAWDALR RSMVHFRGQP VGTIAAVDHA A--EEVLNYD QVFVRDFVPS ALAFLMNGEP DIVKNFLLKT LQLQG----- **StCIN04**

---------- ---------- MVAEAWDALR RTMVHFRGQP VGTIAAVDHA S--EEVLNYD QVFVRDFVPS ALAFLMNGEP DIVKNFLLKT LQLQG----- **SmCIN03**

P-FGQGP--- --------HP MVAEAWDSLR RTLVHFRGQP VGTIAALDNS ---DEKLNYD QVFVRDFVPS ALAFLMNREP EIVKNFLLKT LRLQS----- **SlCIN06**

P-FGQGP--- --------HP MVAEAWDSLR RTLVHFRGQP VGTIAALDNS ---DEKLNYD QVFVRDFVPS ALAFLMNREP EIVKNFLLKT LRLQS----- **SpiCIN06**

P-FGQGP--- --------HP MVAEAWDSLR RTLVHFRGQP VGTIAALDNS ---DEKLNYD QVFVRDFVPS ALAFLMNREP EIVKNFLLKT LRLQS----- **StCIN05**

---------- ---------- ---------- ---------- ---------- ---------- ---------- -----MNREP EIVKNFLLKT LRLQS----- **SpeCIN08**

---------- ---------- ---------- ---------- ---------- ---------- ---------- -----MNREP EIVKNFLLKT LRLQS----- **SmCIN04**

DNYLNGECVS ESPHEKELSE VEKEAWNLLR GAVVNYCGFP VGTGAANDPA D--KQPLNYD QVFIRDFVPS ALAFLLNGEG EIVKNFLLHT LQLQS----- **SlCIN08**

DNYLNGECVS ESPHEKELSE VEKEAWNLLR GAVVNYCGFP VGTGAANDPA D--KQPLNYD QVFIRDFVPS ALAFLLNGEG EIVKNFLLHT LQLQS----- **SpiCIN08**

DNYLNGESVS ESPHEKELSE VEKEAWTLLR GAVVNYCGFP VGTVAANDPA D--MQPLNYD QVFIRDFVPS ALAFLLNGEG GIVKNFLLHT LQLQS----- **StCIN08**

DNYLNGECVS ESPHEKELSE VEKEAWNLLR GAVVNYCGFP VGTVAANDPA D--KQPLNYD QVFIRDFVPS ALAFLLNGEG EIVKNFLLHT LQLQS----- **SpeCIN06**

DNYLNGESVY ESLREKELSE VEKEAWNLLR GAVVNYCGFP IGTVAANDPA D--KQPLNYD QVFIRDFIPS ALAFLLNGEG EIVKNFLLHT LQLQS----- **SmCIN02**

ESVKEGE--- -------ESQ TVKEAWKLLE NAVVKYCGSP IGTLAANDPN D--KLPLNYD QVFIRDFIPS ALAFLLKGEK EIVRNFLLHT LQLQS----- **SlCIN02**

ESVKEGE--- -------ESQ TVKEAWKLLE NAVVKYCGSP IGTLAANDPN D--KLPLNYD QVFIRDFIPS ALAFLLKGEK EIVRNFLLHT LQLQS----- **SpiCIN02**

ESVKEGE--- -------ESQ TVKEAWKLLE NAVVKYCGSP IGTLAANDPN D--KLPLNYD QVFIRDFIPS ALAFLLKGEK EIVRNFLLHT LQLQS----- **SpeCIN02**

ESVKEGE--- -------ESQ TVKEAWRLLE NAVVTYCGSP IGTLAANDPN D--KLPLNYD QVFIRDFIPS ALAFLLKGEK EIVRNFLLHT LQLQS----- **StCIN02**

ESVKEGEGQV -GVQGRGESQ VVKEAWKLLE NAVVSYCGSP IGTLAANDPN D--KLPLNYD QVFIRDFIPS ALAFLLKGEK EIVRNFLLHT LQLQS----- **SmCIN07**

NSRPRTN--- -------TES IEDEAWHFLR AAMVYYCGSP VGTIAANDPS E--ATMLNYD QVFIRDFIPS GIAFLLKGEY DIVRNFILHT LQLQS----- **SlCIN03**

NSRPRTN--- -------TES IEDEAWHFLR AAMVYYCGSP VGTIAANDPS E--ATMLNYD QVFIRDFIPS GIAFLLKGEY DIVRNFILHT LQLQS----- **SpiCIN03**

NSRPRTN--- -------TES IEDEAWHFLR AAMVYYCGSP VGTIAANDPS E--ATMLNYD QVFIRDFIPS GIAFLLKGEY DIVRNFILHT LQLQS----- **SpeCIN03**

NSRPRTN--- -------TES IEDEAWHFLR AAMVYYCGSP VGTIAANDPS E--ATMLNYD QVFIRDFIPS GIAFLLKGEY DIVRNFILHT LQLQS----- **StCIN03**

NSRPRNN--- -------TES IEVSLPAFIL KFMSVVMHIT LG--FHQDP- ---------- ---------- ----CLDGQN YLV---LVAG GRWQS----- **SmCIN05**

DTLNRIA--- -------GNS IEDEAWELLR ESMVYYCGSP VGTIAAKDPT SSTADVLNYD QVFIRDFIPS GIAFLLKGEY EIVRNFILHT LQLQLGMDSW **SlCIN07**

DTLNRIA--- -------GNS IEDEAWELLR ESMVYYCGSP VGTIAAKDPT SSTADVLNYD QVFIRDFIPS GIAFLLKGEY EIVRNFILHT LQLQS----- **SpiCIN07**

DTLNRIA--- -------GNS IEDEAWELLR ESMVYYCGSP VGTIAAKDPT SSTADVLNYD QVFIRDFIPS GIAFLLKGEY EIVRNFILHT LQLQS----- **SpeCIN07**

DTLNRIA--- -------GNS IEDEAWELLR ESMVYYCGSP VGTIAAKDPT SSTADVLNYD QVFIRDFIPS GIAFLLKGEY EIVRNFILHT LQLQS----- **StCIN07**

DPLNRIA--- -------GNS IEDEAWELLR ESMVYYCGSP VGTIAAKDPT SSTANVLNYD QVFIRDFIPS GIAFLLKGEY EIVRNFILHT LQLQS----- **SmCIN06**

**Clustal Conse**

**310 320 330 340 350 360 370 380 390 400**

**....|....| ....|....| ....|....| ....|....| ....|....| ....|....| ....|....| ....|....| ....|....| ....|....|**

---------- REKKIDQFKL GDGVMPASFK VSHDPVR--- --NYETITAD FGESAIGRVA PVDSGFWWII LLRAYTKSTG DTSLAEMPEC QRGIRLILEL **SlCIN01**

---------- REKKIDQFKL GDGVMPASFK VSHDPVR--- --NYETITAD FGESAIGRVA PVDSGFWWII LLRAYTKSTG DTSLAEMPEC QRGIRLILEL **SpiCIN01**

---------- REKKIDQFKL GDGVMPASFK VSHDPVR--- --NYETITAD FGESAIGRVA PVDSGFWWII LLRAYTKSTG DTSLAEMQEC QRGIRLILEL **SpeCIN01**

---------- REKKIDQFKL GDGVMPASFK VSHDPVR--- --NYETITAD FGESAIGRVA PVDSGFWWII LLRAYTKSTG DTSLAEMPEC QRGIRLILEL **StCIN01**

---------- REKKIDQFKL GDGVMPASFK VSHDPVR--- --NYETITAD FGESAIGRVA PVDSGFWWII LLRAYTKSTG DASLAEMPEC QRGIRLILEL **SmCIN01**

---------- ---------- ---------- ---------- ---------- ---------- ---------- ---------- ---------- ---------- **SmCIN08**

---------- WEKRVDRFKL GEGVMPASFK VLHDPVR--- --KTDTIVAD FGESAIGRVA PVDSGFWWII LLRAYTKSTG DLSLSETTEC QKGMRLILSL **SlCIN04**

---------- WEKRVDRFKL GEGVMPASFK VLHDPVR--- --KTDTIVAD FGESAIGRVA PVDSGFWWII LLRAYTKSTG DLSLSETTEC QKGMRLILSL **SpiCIN04**

---------- WEKRVDRFKL GEGVMPASFK VLHDPVR--- --KTDTIVAD FGESAIGRVA PVDSGFWWII LLRAYTKSTG DLSLSETTEC QKGMRLILSL **SpeCIN04**

---------- WEKRVDRFKL GEGVMPASFK VLHDPVR--- --KTDTIVAD FGESAIGRVA PVDSGFWWII LLRAYTKSTG DLSLSETTEC QKGMRLILSL **StCIN06**

---------- WEKRVDRFKL GEGVMPASFK VLHDPVR--- --KTDTIMAD FGESAIGRVA PVDSGFWWII LLRAYTKSTG DVSLAETPEC QRGMRLILSL **SlCIN05**

---------- WEKRVDRFKL GEGVMPASFK VLHDPVR--- --KTDTIMAD FGESAIGRVA PVDSGFWWII LLRAYTKSTG DVSLAETPEC QRGMRLILSL **SpeCIN05**

---------- WEKRVDRFKL GEGVMPASFK VLHDPVR--- --KTDTIMAD FGESAIGRVA PVDSGFWWII LLRAYTKSTG DVSLAETPEC QRGMRLILSL **SpiCIN05**

---------- WEKRVDRFKL GEGVMPASFK VLHDPVR--- --KTDTIMAD FGESAIGRVA PVDSGFWWII LLRAYTKSTG DVSLAETPEC QRGMRLILSL **StCIN04**

---------- WEKKVDRFKL GEGVMPASFK VLHDPVR--- --KTDTIVAD FGESAIGRVA PVDSGFWWII LLRAYTKSTG DLSLSETTEC QKGMRLILSL **SmCIN03**

---------- WEKKIDRFQL GEGVMPASFK VLHDPVR--- --NTETLIAD FGESAIGRVA PIDSGFWWII LLRAYTKSTG DTSLSELPEC QKGMRLILSL **SlCIN06**

---------- WEKKIDRFQL GEGVMPASFK VLHDPVR--- --NTETLIAD FGESAIGRVA PIDSGFWWII LLRAYTKSTG DTSLSELPEC QKGMRLILSL **SpiCIN06**

---------- WEKKIDRFQL GEGVMPASFK VLHDPVR--- --NTETLIAD FGESAIGRVA PIDSGFWWII LLRAYTKSTG DTSLSELPEC QKGMRLILSL **StCIN05**

---------- WEKKIDRFQL GEGVMPASFK VLHDPVR--- --NTETLIAD FGESAIGRVA PIDSGFWWII LLRAYTKSTG DTSLSELPEC QKGMRLILSL **SpeCIN08**

---------- WEKKIDRFQL GEGVMPASFK VLHDPVR--- --NTETLIAD FGESAIGRVA PIDSGFWWII LLRAYTKSTG DTSLSELPEC QKGMRLILSL **SmCIN04**

---------- WEKTVDCYNP GEGLMPASFK VRTVPLDGRN GEFEDMLDPD FGESAIGRVA PVDSGLWWVI LLRAYGRITG DYNLQERVDV QTGICLILNL **SlCIN08**

---------- WEKTVDCYNP GEGLMPASFK VRTVPLDGRN GEFEDMLDPD FGESAIGRVA PVDSGLWWVI LLRAYGRITG DYNLQERVDV QTGICLILNL **SpiCIN08**

---------- WEKSVDCYNP GKGLMPASFK VRTVPLDGSN GEFKDVLDPD FGESAIGRVA PVDSGLWWII LLRAYGRITG DYTLQERVDV QTGICLILHL **StCIN08**

---------- WEKTVDCYNP GEGLMPASFK VRTVPLDGRN GEFEDMLDPD FGESAIGRVA PVDSGLWWII LLRAYGRITG DYTLQERVDV QTGICLILNL **SpeCIN06**

---------- WEKTVDCYKP GEGLMPASFK VRTVPLDGRN DEFEDVLDPD FGESAIGRVA PVDSGLWWII LLRAYGRITG DYTLQERVDV QTGICLILDL **SmCIN02**

---------- WEKTVDCYSP GQGLMPASFK VRTVPLD--D NKYEEVLDPD FGESAIGRVA PVDSGLWWII LLRAYGKITG DYGLQERVDV QTGIKLIINL **SlCIN02**

---------- WEKTVDCYSP GQGLMPASFK VRTVPLD--D NKYEEVLDPD FGESAIGRVA PVDSGLWWII LLRAYGKITG DYGLQERVDV QTGIKLIINL **SpiCIN02**

---------- WEKTVDCYSP GQGLMPASFK VRTVPLD--D NKYEEVLDPD FGESAIGRVA PVDSGLWWII LLRAYGKITG DYGLQERVDV QTGIKLIINL **SpeCIN02**

---------- WEKTVDCYSP GQGLMPASFK VRTVPLD--D NKYEEVLDPD FGESAIGRVA PVDSGLWWII LLRAYGKITG DYGLQERVDV QTGIKLIINL **StCIN02**

---------- WEKTVDCYSP GQGLMPASFK VRTVALD--D NKYEEVLDPD FGESAIGRVA PVDSGLWWII LLRAYGKITG DYGLQERVDV QTGIKLIINL **SmCIN07**

---------- WEKTMDCYSP GQGLMPASFK VRTIPLDNDE SATEDVLDPD FGEAAIGRVA PVDSGLWWII LLRAYGKCSG DLSLQERVDV QTGMKMILRL **SlCIN03**

---------- WEKTMDCYSP GQGLMPASFK VRTIPLDNDE SATEDVLDPD FGEAAIGRVA PVDSGLWWII LLRAYGKCSG DLSLQERVDV QTGMKMILRL **SpiCIN03**

---------- WEKTMDCYSP GQGLMPASFK VRTIPLDNDE SATEDVLDPD FGEAAIGRVA PVDSGLWWII LLRAYGKCSG DLSLQERVDV QTGMKMILRL **SpeCIN03**

---------- WEKTMDCYSP GQGLMPASFK VRTIPLDNDE SATEDVLDPD FGEAAIGRVA PVDSGLWWII LLRAYGKCSG DLSLQERVDV QTGMKMILRL **StCIN03**

---------- WEKTMDCYSP GQGLMPASFK VRTVPLDNDE SATEDVLDPD FGEAAIGRVA PVDSGLWWII LLRAYGKCTG DLSLQERVDV QTGMKMILRL **SmCIN05**

GNFMFITGYS WEKTMDCHSP GQGLMPASFK VRTVPLDGDD SATEEVLDPD FGEAAIGRVA PVDSGLWWII LLRAYGKSSG DLSVQERIDV QTGIKMILRL **SlCIN07**

---------- WEKTMDCHSP GQGLMPASFK VRTVPLDGDD SATEEVLDPD FGEAAIGRVA PVDSGLWWII LLRAYGKSSG DLSVQERIDV QTGIKMILRL **SpiCIN07**

---------- WEKTMDCHSP GQGLMPASFK VRTVPLDGDD SATEEVLDPD FGEAAIGRVA PVDSGLWWII LLRAYGKSSG DLSVQERIDV QTGIKMILRL **SpeCIN07**

---------- WEKTMDCHSP GQGLMPASFK VRTVPLDGDD SATEEVLDPD FGEAAIGRVA PVDSGLWWII LLRAYGKSSG DLSVQERIDV QTGIKMILRL **StCIN07**

---------- WEKTMDCHSP GQGLMPASFK VRTVPLDGDD SATEE----- ---------- ---------- ---------- -----ERIDV QTGIKMILRL **SmCIN06**

**Clustal Conse**

**410 420 430 440 450 460 470 480 490 500**

**....|....| ....|....|....|....| ....|....| ....|....| ....|....| ....|....| .....|....| ....|....| ....|....|**

CLSEGFDTFP TLLCADGCSMIDRRM----- ---------- ---------- ---------- ---------- ---------- -GVYGYPIEI Q--------- **SlCIN01**

CLSEGFDTFP TLLCADGCSMIDRRM----- ---------- ---------- ---------- ---------- ---------- -GVYGYPIEI Q--------- **SpiCIN01**

CLSEGFDTFP TLLCADGCSMIDRRM----- ---------- ---------- ---------- ---------- ---------- -GVYGYPIEI Q--------- **SpeCIN01**

CLSEGFDTFP TLLCADGCSMIDRRM----- ---------- ---------- ---------- ---------- ---------- -GVYGYPIEI Q--------- **StCIN01**

CLSEGFDTFP TLLCADGCCMIDRRM----- ---------- ---------- ---------- ---------- ---------- -GVYGYPIEI Q--------- **SmCIN01**

--YKNFVSY- -------CSQ---------- ---------- ---------- ---------- ---------- ---------- -GVYGYPIEI Q--------- **SmCIN08**

CLSEGFDTFP TLLCADGCSMIDRRM----- ---------- ---------- ---------- ---------- ---------- -GVYGYPIEI Q--------- **SlCIN04**

CLSEGFDTFP TLLCADGCSMIDRRM----- ---------- ---------- ---------- ---------- ---------- -GVYGYPIEI Q--------- **SpiCIN04**

CLSEGFDTFP TLLCADGCSMIDRRM----- ---------- ---------- ---------- ---------- ---------- -GVYGYPIEI Q--------- **SpeCIN04**

CLSEGFDTFP TLLCADGCSMIDRRM----- ---------- ---------- ---------- ---------- ---------- -GVYGYPIEI Q--------- **StCIN06**

CLSEGFDTFP TLLCADGCSMIDRRM----- ---------- ---------- ---------- ---------- ---------- -GIYGYPIEI Q--------- **SlCIN05**

CLSEGFDTFP TLLCADGCSMIDRRM----- ---------- ---------- ---------- ---------- ---------- -GIYGYPIEI Q--------- **SpeCIN05**

CLSEGFDTFP TLLCADGCSMIDRRM----- ---------- ---------- ---------- ---------- ---------- -GIYGYPIEI Q--------- **SpiCIN05**

CLSEGFDTFP TLLCADGCSMIDRRM----- ---------- ---------- ---------- ---------- ---------- -GIYGYPIEI Q--------- **StCIN04**

CLSEGFDTFP TLLCADGCSMIDRRMSIQMM FFLGNTKKAS LPWVRGLQGG ELNPHQQGIM YIPLDLSQLM GSSGGVRKDS LGVYGYPIEI Q--------- **SmCIN03**

CLSEGFDTFP TLLCADGCSMIDRRM----- ---------- ---------- ---------- ---------- ---------- -GVYGYPIEI Q--------- **SlCIN06**

CLSEGFDTFP TLLCADGCSMIDRRM----- ---------- ---------- ---------- ---------- ---------- -GVYGYPIEI Q--------- **SpiCIN06**

CLSEGFDTFP TLLCADGCSMIDRRM----- ---------- ---------- ---------- ---------- ---------- -GVYGYPIEI Q--------- **StCIN05**

CLSEGFDTFP TLLCADGCSMIDRRM----- ---------- ---------- ---------- ---------- ---------- -GVYGYPIEI Q--------- **SpeCIN08**

CLSEGFDTFP TLLCADGCSMIDRRM----- ---------- ---------- ---------- ---------- ---------- -GVYGYPIEI Q--------- **SmCIN04**

CLSDGFDLFP TLLVTDGSCMIDRRM----- ---------- ---------- ---------- ---------- ---------- -GIHGHPLEI Q--------- **SlCIN08**

CLSDGFDLFP TLLVTDGSCMIDRRM----- ---------- ---------- ---------- ---------- ---------- -GIHGHPLEI Q--------- **SpiCIN08**

CLSDGFDMFP TLLVTDGSCMIDRRM----- ---------- ---------- ---------- ---------- ---------- -GIHGHPLEI Q--------- **StCIN08**

CLSDGFDLFP TLLVTDGSCMIDRRM----- ---------- ---------- ---------- ---------- ---------- -GIHGHPLEI Q--------- **SpeCIN06**

CLRDGFDMFP TLLVTDGSCMIDRRM----- ---------- ---------- ---------- ---------- ---------- -GIHGHPLEI Q--------- **SmCIN02**

CLSDGFDMFP SLLVTDGSCMIDRRM----- ---------- ---------- ---------- ---------- ---------- -GIHGHPLEI Q--------- **SlCIN02**

CLSDGFDMFP SLLVTDGSCMIDRRM----- ---------- ---------- ---------- ---------- ---------- -GIHGHPLEI Q--------- **SpiCIN02**

CLSDGFDMFP SLLVTDGSCMIDRRM----- ---------- ---------- ---------- ---------- ---------- -GIHGHPLEI Q--------- **SpeCIN02**

CLSDGFDMFP SLLVTDGSCMIDRRM----- ---------- ---------- ---------- ---------- ---------- -GIHGHPLEI Q--------- **StCIN02**

CLSDGFDMFP SLLVTDGSCMIDRRM----- ---------- ---------- ---------- ---------- ---------- -GIHGHPLEI Q--------- **SmCIN07**

CLADGFDMFP TLLVTDGSCMIDRRM----- ---------- ---------- ---------- ---------- ---------- -GIHGHPLEI Q--------- **SlCIN03**

CLADGFDMFP TLLVTDGSCMIDRRM----- ---------- ---------- ---------- ---------- ---------- -GIHGHPLEI Q--------- **SpiCIN03**

CLADGFDMFP TLLVTDGSCMIDRRM----- ---------- ---------- ---------- ---------- ---------- -GIHGHPLEI Q--------- **SpeCIN03**

CLADGFDMFP TLLVTDGSCMIDRRM----- ---------- ---------- ---------- ---------- ---------- -GIHGHPLEI Q--------- **StCIN03**

CLADGFDMFP TLLVTDGSCMIDRRM----- ---------- ---------- ---------- ---------- ---------- -GIHGHPLEI Q--------- **SmCIN05**

CLADGFDMFP TLLVTDGSCMIDRRM----- ---------- ---------- ---------- ---------- ---------- -GIHGHPLEI Q--------- **SlCIN07**

CLADGFDMFP TLLVTDGSCMIDRRM----- ---------- ---------- ---------- ---------- ---------- -GIHGHPLEI Q--------- **SpiCIN07**

CLADGFDMFP TLLVTDGSCMIDRRM----- ---------- ---------- ---------- ---------- ---------- -GIHGHPLEI Q--------- **SpeCIN07**

CLADGFDMFP TLLVTDGSCMIDRRM----- ---------- ---------- ---------- ---------- ---------- -GIHGHPLEI Q--------- **StCIN07**

CLADGFDMFP TLLVTDGSCMIDRRM----- ---------- ---------- ---------- ---------- ---------- -GIHGHPLEI QEVRIQRHVS **SmCIN06**

..* : .. *::*:*:** * **Clustal Conse**

D188

**510 520 530 540 550 560 570 580 590 600**

**....|....| ....|....| ....|....| ....|....| ....|....| ....|....| ....|....| ....|....| ....|....| ....|....|**

---------- ---------- ---------- ---------- ALFFMALRCA LFLLKHDEEN QECCDAIIKR LHALSFHMRS YYWLDIKQLN DIYRYKTEEY **SlCIN01**

---------- ---------- ---------- ---------- ALFFMALRCA LFLLKHDEEN QECCDAIIKR LHALSFHMRS YYWLDIKQLN DIYRYKTEEY **SpiCIN01**

---------- ---------- ---------- ---------- ALFFMALRCA LFLLKHDEEN QECCDAIIKR LHALSFHMRS YYWLDIKQLN DIYRYKTEEY **SpeCIN01**

---------- ---------- ---------- ---------- ALFFMALRCA LFLLKHDEEN RECCDAIIKR LHALSFHMRS YYWLDIKQLN DIYRYKTEEY **StCIN01**

---------- ---------- ---------- ---------- ALFFMALRCA LLLLKHDEEN RECCDAIIKR LHALSFHMRS YFWLDIKQLN DIYRYKTEEY **SmCIN01**

---------- ---------- ---------- ---------- ALFFMALRCA LLLLKHDEEN RECCDAIIKR LHALSFHMRS YFWLDIKQLN DIYRYKTEEY **SmCIN08**

---------- ---------- ---------- ---------- ALFFMALRSA LAMLKHDTEG KEFIERIVKR LHALSYHMRS YFWLDFQQLN DIYRYKTEEY **SlCIN04**

---------- ---------- ---------- ---------- ALFFMALRSA LAMLKHDTEG KEFIERIVKR LHALSYHMRS YFWLDFQQLN DIYRYKTEEY **SpiCIN04**

---------- ---------- ---------- ---------- ALFFMALRSA LAMLKHDTEG KEFIERIVKR LHALSYHMRS YFWLDFQQLN DIYRYKTEEY **SpeCIN04**

---------- ---------- ---------- ---------- ALFFMALRSA LAMLKHDTEG KEFIERIVKR LHALSYHMRS YFWLDFQQLN DIYRYKTEEY **StCIN06**

---------- ---------- ---------- ---------- ALFFMALRSA LAMLKHDTEG GEFVERIVKR LHALSYHMRS YFWLDFQQLN DIYRYKTEEY **SlCIN05**

---------- ---------- ---------- ---------- ALFFMALRSA LAMLKHDTEG GEFVERIVKR LHALSYHMRN YFWLDFQQLN DIYRYKTEEY **SpeCIN05**

---------- ---------- ---------- ---------- ALFFMALRSA LAMLKHDTEG GEFVERIVKR LHALSYHMRS YFWLDFQQLN DIYRYKTEEY **SpiCIN05**

---------- ---------- ---------- ---------- ALFFMALRSA LAMLKHDTEG GEFVERIVKR LHALSYHMRS YFWLDFQQLN DIYRYKTEEY **StCIN04**

---------- ---------- ---------- ---------- ALFFMALRSA LAMLKPDAEG KDFIERIVKR LHALSYHMRS YFWLDFQQLN DIYRYKTEEY **SmCIN03**

---------- ---------- ---------- ---------- ALFFMALRCA LLLLKHDAEG KEFVERIVKR LHALSYHMRN YFWLDLKQLN DIYRYKTEEY **SlCIN06**

---------- ---------- ---------- ---------- ALFFMALRCA LLLLKHDAEG KEFVERIVKR LHALSYHMRN YFWLDLKQLN DIYRYKTEEY **SpiCIN06**

---------- ---------- ---------- ---------- ALFFMALRCA LLLLKHDAEG KEFVERIVKR LHALSYHMRN YFWLDLKQLN DIYRYKTEEY **StCIN05**

---------- ---------- ---------- ---------- ALFFMALRCA LLLLKHDAEG KEFVERIVKR LHALSYHMRN YFWLDLKQLN DIYRYKTEEY **SpeCIN08**

---------- ---------- ---------- ---------- ALFFMALRCA LVLLKHDAEG KEFVERIVKR LHALSYHMRS YFWLDLKQLN DIYRYKTEEY **SmCIN04**

---------- ---------- ---------- ---------- ALFYSALRSS REMLSINDST KSLVSAINNR LSALSFHMRD YYWLDRKKIN EIYRYKTEEY **SlCIN08**

---------- ---------- ---------- ---------- ALFYSALRSS REMLSINDST KSLVSAINNR LSALSFHMRD YYWLDRKKIN EIYRYKTEEY **SpiCIN08**

---------- ---------- ---------- ---------- ALFYSALRSS HEMLTINDST KSLVSAINNR LSALSFHMRE YYWLDRKKIN EIYRYKTEEY **StCIN08**

---------- ---------- ---------- ---------- ALFYSALRSS REMLSINDST KSLVSAINNR LSALSFHMRE YYWLDRKKIN EIYRYKTEEY **SpeCIN06**

---------- ---------- ---------- ---------- ALFYSALRSS REMLTINDST KSLVAAINNR LSALSFHMRE YYWLDRKKIN EIYRYKTEEY **SmCIN02**

---------- ---------- ---------- ---------- ALFYSALRCS HELLSLDDGS KNLVNAINNR LSALSFHIRE YYWVDMKKIN EIYRYKTEEY **SlCIN02**

---------- ---------- ---------- ---------- ALFYSALRCS HELLSLDDGS KNLVNAINNR LSALSFHIRE YYWVDMKKIN EIYRYKTEEY **SpiCIN02**

---------- ---------- ---------- ---------- ALFYSALRCS RELLSLDEGS KNLVNAINNR LSALSFHIRE YYWVDMKKIN EIYRYKTEEY **SpeCIN02**

---------- ---------- ---------- ---------- ALFYSALRCS RELLSLDEGS KNLVNAINNR LSALSFHIRE YYWVDMKKIN EIYRYKTEEY **StCIN02**

---------- ---------- ---------- ---------- ALFYSALRCS RELLSLDEGS KNLVNTINNR LSALSFHIRE YYWVDMKKIN EIYRYKTEEY **SmCIN07**

---------- ---------- ---------- ---------- ALYYSALLGA REMLAPEEAS TDLVRALNNR LLALSFHIRE YYWIDVKKLN EIYRYKTEEY **SlCIN03**

---------- ---------- ---------- ---------- ALYYSALLGA REMLAPEEAS TDLVRALNNR LLALSFHIRE YYWIDVKKLN EIYRYKTEEY **SpiCIN03**

---------- ---------- ---------- ---------- ALYYSALLGA REMLAPEEAS TDLVRALNNR LLALSFHIRE YYWIDVKKLN EIYRYKTEEY **SpeCIN03**

---------- ---------- ---------- ---------- ALYYSALLGA REMLAPEEAS TDLVRALNNR LLALSFHIRE YYWIDVKKLN EIYRYKTEEY **StCIN03**

---------- ---------- ---------- ---------- ALYYSALLGA REMLAPEEAS TDLVRALNNR LLALSFHIRE YYWIDVKKLN EIYRYKTEEY **SmCIN05**

---------- ---------- ---------- ---------- ALFHSALLCA REMLTPEDGS ADLIRALNNR LVALSFHIRE YYWIDMKKLN EIYRYQTEEY **SlCIN07**

---------- ---------- ---------- ---------- ALFHSALLCA REMLTPEDGS ADLIRALNNR LVALSFHIRE YYWIDMKKLN EIYRYQTEEY **SpiCIN07**

---------- ---------- ---------- ---------- ALFHSALLCA REMLTPEDGS ADLIRALNNR LVALSFHIRE YYWIDMKKLN EIYRYQTEEY **SpeCIN07**

---------- ---------- ---------- ---------- ALFHSALLCA REMLTPEDGS ADLIRALNNR LVALSFHIRE YYWIDMKKLN EIYRYQTEEY **StCIN07**

YKLLRSLTSV KGWEIGLLTH PNLFFTGVGS PLISVHAPVE ALFHSALLCA REMLAPEDGS ADLIRALNNR LVALSFHIRE YYWIDMRKLN EIYRYQTEEY **SmCIN06**

**:. ** : :* : . : :* * ***:*:*. *:*:* :::* :****:**** **Clustal Conse**

**610 620 630 640 650 660 670 680 690 700**

**....|....| ....|....| ....|....| ....|....| ....|....| ....|....| ....|....| ....|....| ....|....| ....|....|**

SHTAVNKFNV MPDSLPEWVF DFMPTRGGYF IGNVSPAHMD FRWFCLGNCI SILSSLATPE QASAIMDLVE SRWQELVGEM PLKICYPAME GHEWRIVTGC **SlCIN01**

SHTAVNKFNV MPDSLPEWVF DFMPTRGGYF IGNVSPAHMD FRWFCLGNCI SILSSLATPE QASAIMDLVE SRWQELVGEM PLKICYPAME GHEWRIVTGC **SpiCIN01**

SHTAVNKFNV MPDSLPEWVF DFMPTRGGYF IGNVSPAHMD FRWFCLGNCI SILSSLATPE QASAIMDLVE SRWQELVGEM PLKICYPAME GHEWRIVTGC **SpeCIN01**

SHTAVNKFNV MPDSLPEWVF DFMPTRGGYF IGNVSPAHMD FRWFCLGNCI SILSSLATPE QASAIMDLVE SRWQELVGEM PLKICYPAME GHEWRIVTGC **StCIN01**

SHTAVNKFNV MPDSLPEWVF DFMPSRGGYF IGNVSPAHMD FRWFCLGNCI AILSSLATPE QASAIMDLVE SRWQELVGEM PLKICYPAME GHEWRIVTGC **SmCIN01**

SHTAVNKFNV MPDSLPEWVF DFMPSRGGYF IGNVSPAHMD FRWFCLGNCI AILSSLATPE QASAIMDLVE SRWQELVGEM PLKICYPAME GHEWRIVTGC **SmCIN08**

SHTAVNKFNV IPDSIPEWVF DFVPTRGGYF IGNVSPARMD FRWFALGNCI AILSSLATPE QASAIMDLIE SRWEELVADM PLKICYPAIE NHEWRIVTGC **SlCIN04**

SHTAVNKFNV IPDSIPEWVF DFVPTRGGYF IGNVSPARMD FRWFALGNCI AILSSLATPE QASAIMDLIE SRWEELVADM PLKICYPAIE NHEWRIVTGC **SpiCIN04**

SHTAVNKFNV IPDSIPEWVF DFVPTRGGYF IGNVSPARMD FRWFALGNCI AILSSLATPE QASAIMDLIE SRWEELVADM PLKICYPAIE NHEWRIVTGC **SpeCIN04**

SHTAVNKFNV IPDSIPEWVF DFVPTRGGYF IGNVSPARMD FRWFALGNCI AILSSLATPE QASAIMDLIE ARWEELVADM PLKICYPAIE NHEWRIVTGC **StCIN06**

SHTAVNKFNV IPDSIPDWVF EFVPKRGGYF VGNVSPARMD FRWFALGNCI AILSSLATPE QASAIMDLIE ARWDELVAEM PLKISYPALE NHDWRHITGC **SlCIN05**

SHTAVNKFNV IPDSIPDWVF EFVPKRGGYF IGNVSPARMD FRWFALGNCI AILSSLATPE QASAIMDLIE ARWDELVAEM PLKISYPALE NHDWRHITGC **SpeCIN05**

SHTAVNKFNV IPDSIPDWVF EFVPKRGGYF VGNVSPARMD FRWFALGNCI AILSSLATPE QASAIMDLIE ARWDELVAEM PLKISYPALE NHDWRHITGC **SpiCIN05**

SHTAVNKFNV IPDSIPDWVF EFVPKRGGYF IGNVSPARMD FRWFALGNCI AILSSLATPE QASAIMDLIE ARWDELVAEM PLKISYPALE NHDWRLITGC **StCIN04**

SHTAVNKFNV IPDSIPEWVF DFVPTCGGYF IGNVSPARMD FRWFALGNCI AIMSSLATPE QASAIMDLIE SRWEELVADM PLKICYPAIE NHEWRIVTGC **SmCIN03**

SHTAVNKFNV MPDSLPEWVF DFMPVSGGYF LGNVGPSNMD FRWFCLGNCI AILSSLATPE QATKIMDLID SRWHELVGEM PLKVCYPAIE GHEWRIVTGC **SlCIN06**

SHTAVNKFNV MPDSLPEWVF DFMPVSGGYF LGNVGPSNMD FRWFCLGNCI AILSSLATPE QATKIMDLID SRWHELVGEM PLKVCYPAIE GHEWRIVTGC **SpiCIN06**

SHTAVNKFNV MPDSLPEWIF DFMPVSGGYF LGNVGPSNMD FRWFCLGNCI AILSSLATPE QATKIMELIE SRWHELVGEM PLKVCYPAIE GHEWRIVTGC **StCIN05**

SHTAVNKFNV MPDSLPEWVF DFMPVSGGYF LGNVGPSNMD FRWFCLGNCI AILSSLATPE QATKIMDLID SRWHELVGEM PLKVCYPAIE GHEWRIVTGC **SpeCIN08**

SHTAVNKFNV MPDSLPEWVF DFMPVSGGYF LGNVGPSNMD FRWFCLGNCI AILSCLATPE QATKIMDLIE SRWHELVGEM PLKVCYPAIE GHEWRIVTGC **SmCIN04**

STDAINKFNI YPDQIPSWLV DWIPEIGGYL VGNLQPAHMD F--------- ---------- ---------- HKWDDLMGKM PLKICYPALE HEEWRIITGS **SlCIN08**

STDAINKFNI YPDQIPSWLV DWIPEIGGYL VGNLQPAHMD F--------- ---------- ---------- HKWDDLMGKM PLKICYPALE HEEWRIITGS **SpiCIN08**

STDAINKFNI YPDQIPSWLM DWIPEIGGYL VGNLQPAHMD FRFFTHGNIW TIISSLGSHE QNESILNLIE DKWDDLMGKM PLKICYPALE HEEWCIITGS **StCIN08**

STDAINKFNI YPDQIPSWLV DWIPEIGGYL VGNLQPAHMD FRFFTHGNIW TIISSLGSHE QNESILNLIE DKWDDLMGKM PLKICYPALE HEEWRIITGS **SpeCIN06**

STDAINKFNI YPDQIPSWLV DWIPEIGGYL VGNLQPAHMD FRFFTHGNIW TIISSLGSHE QNESILNLIE DKWDDLMGKM PLKICYPALE HEEWRIITGS **SmCIN02**

STEATNKFNI YPEQIPHWLM DWIPEEGGYL IGNLQPAHMD FRFFTLGNLW SIVSSLSTPK QNEAILNLIE AKWYDLVGLM PLKICYPALE SEDWRIITGS **SlCIN02**

STEATNKFNI YPEQIPHWLM DWIPEEGGYL IGNLQPAHMD FRFFTLGNLW SIVSSLSTPK QNEAILNLIE AKWYDLVGLM PLKICYPALE SEDWRIITGS **SpiCIN02**

STEATNKFNI YPEQIPHWLM DWIPEEGGYL IGNLQPAHMD FRFFTLGNLW SIVSSLSTPK QNEAILNLIE AKWYDLVGLM PLKICYPALE SEDWRIITGS **SpeCIN02**

STEATNKFNI YPEQIPHWLM DWIPEEGGYL IGNLQPAHMD FRFFTLGNLW SIVSSLSTPK QNEAILNLIE AKWYDLVGLM PLKICYPALE SEDWRIITGS **StCIN02**

STEATNKFNI YPEQIPHWLM DWIPEEGGYL IGNLQPAHMD FRFFTLGNLW SIVSSLGTPK QNEAILNLIE AKWYDLVGLM PLKICYPALE SEDWRIITGS **SmCIN07**

SYDAINKFNI YPDQIPPWLV EWMPSEGGYL IGNLQPAHMD FRFFSLGNVW SIVSSLANID QSHAILDLIE AKWEDLVADM PLKICYPALE GQEWRIITGG **SlCIN03**

SYDAINKFNI YPDQIPPWLV EWMPSEGGYL IGNLQPAHMD FRFFSLGNVW SIVSSLANID QSHAILDLIE AKWEDLVADM PLKICYPALE GQEWRIITGG **SpiCIN03**

SYDAINKFNI YPDQIPPWLV EWMPSEGGYL IGNLQPAHMD FRFFSLGNVW SIVSSLANID QSHAILDLIE AKWEDLVADM PLKICYPALE GQEWRIITGG **SpeCIN03**

SYDAINKFNI YPDQIPPWLV EWMPSEGGYL IGNLQPAHMD FRFFSLGNVW SIVSSLANID QSHAILDLIE AKWEDLVADM PLKICYPALE GQEWRIITGG **StCIN03**

SYDAINKFNI YPDQIPPWLV EWMPSEGGYL IGNLQPAHMD FRFFSLGNIW SIVSSLANID QSHAILDLIE AKWEELVADM PLKICYPALE GQEWRIITGG **SmCIN05**

SYDAVNKFNI YPDQISPWLV DWMPSKGGYL IGNLQPAHMD FRFFSLGNLW SIVCSLTTDD QSHAILDLIE AKWTDLVADM PFKICYPALE GQEWKIITGC **SlCIN07**

SYDAVNKFNI YPDQISPWLV DWMPSKGGYL IGNLQPAHMD FRFFSLGNLW SIVCSLTTDD QSHAILDLIE AKWTDLVADM PFKICYPALE GQEWKIITGC **SpiCIN07**

SYDAVNKFNI YPDQISPWLV DWMPSKGGYL IGNLQPAHMD FRFFSLGNLW SIVCSLTTDD QSHAILDLIE AKWTDLVADM PFKICYPALE GQEWKIITGC **SpeCIN07**

SYDAVNKFNI YPDQISPWLV DWMPSKGGYL IGNLQPAHMD FRFFSLGNLW SIVCSLTTDD QSHAILDLIE AKWTDLVADM PFKICYPALE GQEWKIITGC **StCIN07**

SYDAVNKFNI YPDQIPPWLV EWMPSKGGYL IGNLQPAHMD FRFFSLGNLW SIVCSLATDD QSHAILDLIE AKWTDLVADM PFKICYPALE GQEWKIITGC **SmCIN06**

* * ****: *:.:. *:. :::* ***: :**: *:.** * :* :*:. * *:*:.***:* .:* :** **Clustal Conse**

**710 720 730 740 750 760 770 780 790 800**

**....|....| ....|....| ....|....| ....|....| ....|....| ....|....| ....|....| ....|....| ....|....| ....|....|**

DPKNTSWSYH NGGTWPVLLW LLTAAAIKTG RPQIARRAIE LAESRLLKDS WPEYYDGKLG RFIGKQARKF QTWSIAGYLV ARMMLEDPSH LGMISLEEDK **SlCIN01**

DPKNTSWSYH NGGTWPVLLW LLTAAAIKTG RPQIARRAIE LAESRLLKDS WPEYYDGKLG RFIGKQARKF QTWSIAGYLV ARMMLEDPSH LGMISLEEDK **SpiCIN01**

DPKNTSWSYH NGGTWPVLLW LLTAAAIKTG RPQIARRAIE LAESRLLKDS WPEYYDGKLG RFIGKQARKF QTWSIAGYLV ARMMLEDPSH LGMISLEEDK **SpeCIN01**

DPKNTSWSYH NGGTWPVLLW LLTAAAIKTG RPQIARRAIE LAESRLLKDS WPEYYDGKLG RFIGKQARKF QTWSIAGYLV ARMMLEDPSH LGMISLEEDK **StCIN01**

DPKNTSWSYH NGGTWPVLLW LLTAAAIKTG RPQIARRAIE LAESRLLKDN WPEYYDGKLG RFIGKQARKF QTWSIAGYLV ARMMLEDPSH LGMIALEEDK **SmCIN01**

DPKNTSWSYH NGGTWPVLLW LLTAAAIKTG RPQIARRAIE LAESRLLKDN WPEYYDGKLG RFIGKQARKF QTWSIAGYLV ARMMLEDPSH LGMIALEEDK **SmCIN08**

DPKNIRWSYH NGGSWPVLLW LLTAACIKTG RPQIARRAID LAESRLLKDS WPEYYDGKLG RYIGKQARKH QTWSIAGYLV AKMLLEDPSH LGMISLEEDK **SlCIN04**

DPKNIRWSYH NGGSWPVLLW LLTAACIKTG RPQIARRAID LAESRLLKDS WPEYYDGKLG RYIGKQARKH QTWSIAGYLV AKMLLEDPSH LGMISLEEDK **SpiCIN04**

DPKNIRWSYH NGGSWPVLLW LLTAACIKTG RPQIARRAID LAESRLLKDS WPEYYDGKLG RYIGKQARKH QTWSIAGYLV AKMLLEDPSH LGMISLEEDK **SpeCIN04**

DPKNIRWSYH NGGSWPVLLW LLTAACIKTG RPQIARRAID LAESRLLKDS WPEYYDGKLG RYIGKQARKH QTWSIAGYLV AKMLLEDPSH LGMISLEEDK **StCIN06**

DPKNTRWSYH NGGSWPVLLW LLTAACIKTG RPQIARRAID LAESRLSKDS WPEYYDGTVG RYIGKQARKY QTWSIAGYLV AKMLLEDPSH LGMIALEEDK **SlCIN05**

DPKNTRWSYH NGGSWPVLLW LLTAACIKTG RPQIARRAID LAESRLSKDS WPEYYDGTVG RYIGKQARKY QTWSIAGYLV AKMLLEDPSH LGMIALEEDK **SpeCIN05**

DPKNTRWSYH NGGSWPVLLW LLTAACIKTG RPQIARRAID LAESRLSKDS WPEYYDGTVG RYIGKQARKY QTWSIAGYLV AKMLLEDPSH LGMIALEEDK **SpiCIN05**

DPKNTRWSYH NGGSWPVLLW LLTAACIKTG RPQIARRAID LAESRLSKDS WPEYYDGTVG RYIGKQARKY QTWSIAGYLV AKMLLEDPSH LGMIALEEDK **StCIN04**

DPKNIRWSYH NGGSWPVLLW LLTAACIKTG RPQIARRAID LAESRLLKDG WPEYYDGKLG RYIGKQARKH QTWSIAGYLV AKMLLEDPSH LGMISLEEDK **SmCIN03**

DPKNTRWSYH NGGSWPVLLW LLTAACIKTG RPQIARRAIE LAEQRLSKDG WPEYYDGKLG RFIGKQARKH QTWSIAGYLV AKMMLEDPSH LGMISLEEDK **SlCIN06**

DPKNTRWSYH NGGSWPVLLW LLTAACIKTG RPQIARRAIE LAEQRLSKDG WPEYYDGKLG RFIGKQARKH QTWSIAGYLV AKMMLEDPSH LGMISLEEDK **SpiCIN06**

DPKNTRWSYH NGGSWPVLLW LLTAACIKTG RPQLARRAIE VAEQRLSKDG WPEYYDGKLG RFIGKQARKY QTWSIAGYLV AKMMLEDPSH LGMISLEEDK **StCIN05**

DPKNTRWSYH NGGSWPVLLW LLTAACIKTG RPQIARRAIE LAEQRLSKDG WPEYYDGKLG RFIGKQARKH QTWSIAGYLV AKMMLEDPSH LGMISLEEDK **SpeCIN08**

DPKNTRWSYH NGGSWPVLLW LLTAACIKTG RPQIARRAIE LAEQRLSKDG WPEYYDGKLG RFIGKQARKY QTWSITGYLV AKMMLEDPSH LGMISLEEDK **SmCIN04**

DPKNTPWSYH NGGSWPTLLW QFTLACIKMG RPELAQKAVD LAEKRLSADH WPEYYDTRHG RFIGKQARLC QTWTIAGYLT SKMLLQNPDM ASKLFWNEDY **SlCIN08**

DPKNTPWSYH NGGSWPTLLW QFTLACIKMG RPELAQKAVD LAEKRLSADH WPEYYDTRHG RFIGKQARLC QTWTIAGYLT SKMLLQNPDM ASKLFWNEDY **SpiCIN08**

DPKNTPWSYH NGGSWPTLLW QFTLACIKMG RPELAQKAVD LAEKRLSADH WPEYYDTRHG RFIGKQARLC QTWTIAGYLT SKMLLQNPDM ASKLFWNEDY **StCIN08**

DPKNTPWSYH NGGSWPTLLW QFTLACIKMG RPELAQKAVD LAEKRLSADH WPEYYDTRHG RFIGKQARLC QTWTIAGYLT SKMLLQNPDM ASKLFWNEDY **SpeCIN06**

DPKNTPWSYH NGGSWPTLLW QFTLACIKMG RPELAKKAVD LAEKNLSADH WPEYYDTRHG RFIGKQARLH QTWTIAGYLT SKMLLKNPDV ASKLFWNEDY **SmCIN02**

DPKNTPWSYH NGGSWPTLLW QFTLACIKMN RLDLAKKAVD SAEKRLGVDQ WPEYYDTRYG KFTGKQARLY QTWTIAGFLT SKMLLENPET ASLLFWEEDY **SlCIN02**

DPKNTPWSYH NGGSWPTLLW QFTLACIKMN RLDLAKKAVD SAEKRLGVDQ WPEYYDTRYG KFTGKQARLY QTWTIAGFLT SKMLLENPET ASLLFWEEDY **SpiCIN02**

DPKNTPWSYH NGGSWPTLLW QFTLACIKMN RLDLAKKAVD SAEKRLGVDQ WPEYYDTRYG KFTGKQARLY QTWTIAGFLT SKMLLENPET ASLLFWEEDY **SpeCIN02**

DPKNTPWSYH NGGSWPTLLW QFTLACIKMN RLDLAKKAVD SAEKRLRVDQ WPEYYDTRYG KFTGKQARLY QTWTIAGFLT SKMLLENPET ASLLFWEEDY **StCIN02**

DPKNT----- ---------- ---------- ---------- ---------- ---------- ---------- ---------- ---------- ---------- **SmCIN07**

DPKNTPWSYH NGGSWPTLLW QLTVACIKMK RPEIAEKAIK IAERRLSRDR WPEYYDTRRG GFIGKQARLF QTWTIAGYLV AKLLIANPEA AKMVINVEDT **SlCIN03**

DPKNTPWSYH NGGSWPTLLW QLTVACIKMK RPEIAEKAIK IAERRLSRDR WPEYYDTRRG GFIGKQARLF QTWTIAGYLV AKLLIANPEA AKMVINVEDT **SpiCIN03**

DPKNTPWSYH NGGSWPTLLW QLTVACIKMK RPEIAEKAIK IAERRLSRDR WPEYYDTRRG GFIGKQARLF QTWTIAGYLV AKLLIANPEA AKMVINVEDT **SpeCIN03**

DPKNTPWSYH NGGSWPTLLW QLTVACIKMK RPEIAEKAIK IAERRLSRDR WPEYYDTRRG GFIGKQARLF QTWTIAGYLV AKLLIANPEA AKMVINVEDT **StCIN03**

DPKNTPWSYH NGGSWPTLLW QLTVACIKMK RPEIAEKAIK TAERRLSRDR WPEYYDTRRG GFIGKQARLF QTWTIAGYLV AKLLIADPEA AKMVIKVEDT **SmCIN05**

DPKNTPWSYH NGGSWPTLLW QLAVASIKMN RPEIAAKAVE VAEKRISQDK WPEYYDTKKA RFIGKQARLF QTWSIAGYLV AKLLLANPSS AKILISQEDS **SlCIN07**

DPKNTPWSYH NGGSWPTLLW QLAVASIKMN RPEIAAKAVE VAEKRISQDK WPEYYDTKKA RFIGKQARLF QTWSIAGYLV AKLLLANPSS AKILISQEDS **SpiCIN07**

DPKNTPWSYH NGGSWPTLLW QLAVASIKMN RPEIAAKAVE VAEKRISQDK WPEYYDTKKA RFIGKQARLF QTWSIAGYLV AKLLLANPSS AKILISQEDS **SpeCIN07**

DPKNTPWSYH NGGAWPTLLW QLAVASIKMN RPEIAAKAVE VAEKRISRDK WPEYYDTKKA RFIGKQARLY QTWSIAGYLV AKLLLANPSA AKILISQEDS **StCIN07**

DPKNTPWSYH NGGAWPTLLW QLAVACIKMN RPEIGAKAVE VAEKRISRDK WPEYYDTKKA RFIGKQARLF QTWSIAGYLV AKLLLANPSA AKLLVTQEDP **SmCIN06**

**** **Clustal Conse**

E414

Arg430

**810 820 830 840 850**

**....|....| ....|....| ....|....| ....|....| ....|....| ....**

QMKPTMKRSA ---------- ---------- ---------- ----SWTC-- ----  **SlCIN01**

QMKPTMKRSA ---------- ---------- ---------- ----SWTC-- ----  **SpiCIN01**

QMKPTMKRSA ---------- ---------- ---------- ----SWTC-- ----  **SpeCIN01**

QMKPTMKRSA ---------- ---------- ---------- ----SWTC-- ----  **StCIN01**

QMKPTMKRSA ---------- ---------- ---------- ----SWTC-- ----  **SmCIN01**

QMKPTMKRSA ---------- ---------- ---------- ----SWTC-- ----  **SmCIN08**

QMKPVIKRSS ---------- ---------- ---------- ----SWTF-- ----  **SlCIN04**

QMKPVIKRSS ---------- ---------- ---------- ----SWTF-- ----  **SpiCIN04**

QMKPVIKRSS ---------- ---------- ---------- ----SWTF-- ----  **SpeCIN04**

QMKHVIKRSS ---------- ---------- ---------- ----SWTF-- ----  **StCIN06**

QMKPVIKRSA ---------- ---------- ---------- ----SWTC-- ----  **SlCIN05**

QMKPVIKRSA ---------- ---------- ---------- ----SWTC-- ----  **SpeCIN05**

QMKPVIKRSA ---------- ---------- ---------- ----SWTC-- ----  **SpiCIN05**

QMKPVIKRSA ---------- ---------- ---------- ----SWTC-- ----  **StCIN04**

QMKPVIKRSS ---------- ---------- ---------- ----SWTC-- ----  **SmCIN03**

QLKPVLKRSA ---------- ---------- ---------- ----SF---- ----  **SlCIN06**

QLKPVLKRSA ---------- ---------- ---------- ----SF---- ----  **SpiCIN06**

QLKPVLKRSA ---------- ---------- ---------- ----SF---- ----  **StCIN05**

QLKPVLKRSA ---------- ---------- ---------- ----SF---- ----  **SpeCIN08**

QLKPVLKRSA ---------- ---------- ---------- ----SF----- ---  **SmCIN04**

ELLENCVCAL RPNGRRKCSR S--------- ---------- -AARSQVGL- ----  **SlCIN08**

ELLENCVCAL RPNGRRKCSR S--------- ---------- -AARSQVGL- ----  **SpiCIN08**

ELLENCVCAL RPNGRRKCSR S--------- ---------- -ATRSQVGL- ----  **StCIN08**

ELLENCVCAL RPNGRRKCLR S--------- ---------- -AARSQVGL- ----  **SpeCIN06**

ELLENCVCAL KANGSRKCSR Y--------- ---------- -SARSQVGL- ----  **SmCIN02**

DLLENCVCAL KKSGRKKCSR G--------- ---------- -AAKSQILV- ----  **SlCIN02**

DLLENCVCAL KKSGRKKCSR G--------- ---------- -AAKSQILV- ----  **SpiCIN02**

DLLEICVCAL KKSGRKKCSR G--------- ---------- -AAKSQILV- ----  **SpeCIN02**

DLLEICVCAL KKSGRKKCSR G--------- ---------- -AAKSQILV- ----  **StCIN02**

---------- ---------- ---------- ---------- ---------- ----  **SmCIN07**

ELLSAFSSIL ---------- ---------- ---------- ----SSNPRRKRSR-  **SlCIN03**

ELLSAFSSIL ---------- ---------- ---------- ----SSNPRRKRSR-  **SpiCIN03**

ELLSAFSSIL ---------- ---------- ---------- ----SSNPRRKRSR-  **SpeCIN03**

ELLSAFSSIL ---------- ---------- ---------- ----SSNPRRKRSR-  **StCIN03**

ELLSAFSSIL ---------- ---------- ---------- ----SSIQRLQRWE-  **SmCIN05**

ELLNAFSCAI ---------- ---------- ---------- ----SSNPRRKKRG-  **SlCIN07**

ELLNAFSCAI ---------- ---------- ---------- ----SSNPRRKKRG-  **SpiCIN07**

ELLNAFSCAI ---------- ---------- ---------- ----SSNPRRKKRG-  **SpeCIN07**

ELLNAFSCAI ---------- ---------- ---------- ----SSNPRRKKRG-  **StCIN07**

ELLNAFSCAI ---------- ---------- ---------- ----SSNPRRKKRG-  **SmCIN06**

**Clustal Conse**

Ser547
