# Supplementary material for: Evolutionary Conservation and Expression Patterns of Neutral/Alkaline Invertases in Solanum
Source: Biomolecules. 2019 Nov 21;9(12):763. doi: 10.3390/biom9120763 (PMC6995568; doi:10.3390/biom9120763)
Supplement: Supplementary file 1 [file biomolecules-09-00763-s001.zip › Supplemental Figure S1.docx]

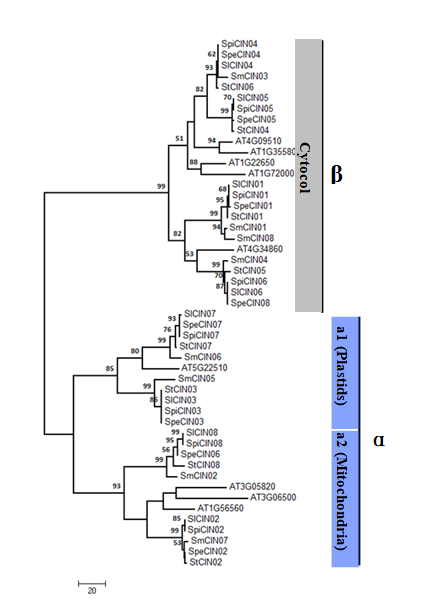


Supplemental Figure S1 Phylogenetic relationship of *Solanum* CINs with *Arabidopsis*. Phylogenetic tree of CIN proteins was constructed with the maximum-parsimony method using MEGA 7.0 software. Support values shown in each line in the phylogenetic tree. Putative CIN genes divided into two subfamilies (α and β) on the basis of their *in silico* prediction of subcellular localization; α subfamily further divided into α1 and α2.
